# Performance of the Use of Genetic Information to Assess the Risk of Colorectal Cancer in the Basque Population

**DOI:** 10.3390/cancers14174193

**Published:** 2022-08-29

**Authors:** Koldo Garcia-Etxebarria, Ane Etxart, Maialen Barrero, Beatriz Nafria, Nerea Miren Segues Merino, Irati Romero-Garmendia, Andre Franke, Mauro D’Amato, Luis Bujanda

**Affiliations:** 1 Biodonostia, Gastrointestinal Genetics Group, 20014 San Sebastián, Spain; 2 Centro de Investigación Biomédica en Red de Enfermedades Hepáticas y Digestivas (CIBERehd), 08036 Barcelona, Spain; 3 Biodonostia, Gastrointestinal Disease Group, Universidad del País Vasco (UPV/EHU), 20014 San Sebastián, Spain; 4 Department of Genetics, Physical Anthropology and Animal Physiology, University of the Basque Country (Universidad del País Vasco/Euskal Herriko Unibertsitatea), 48940 Leioa, Spain; 5 Institute of Clinical Molecular Biology, Christian-Albrechts-University of Kiel, 24105 Kiel, Germany; 6 Gastrointestinal Genetics Lab, CIC bioGUNE, Basque Research and Technology Alliance, 48160 Derio, Spain; 7 IKERBASQUE, Basque Foundation for Sciences, 48009 Bilbao, Spain; 8 Department of Medicine and Surgery, LUM University, 70010 Casamassima, Italy

**Keywords:** colorectal cancer, genome-wide association study, Mendelian randomization, polygenic risk scores

## Abstract

**Simple Summary:**

The risk of developing colorectal cancer (CRC) is partially associated with genetics. Different studies have provided valuable genetic information to understand the biology behind CRC and to build models of genetic risk. However, the study of the applicability of such genetic information within the Basque population is limited. Thus, our objectives were to find out if the genetic variants associated with CRC in other populations are the same in the Basque population and to assess the performance of the use of genetic information to calculate the risk of developing CRC. We found that the available genetic information can be applied to the Basque population, although local genetic variation can affect its use. Our findings will help to refine the use of CRC genetic risk calculation in the Basque population, and we expect that our findings could be useful for other populations.

**Abstract:**

Although the genetic contribution to colorectal cancer (CRC) has been studied in various populations, studies on the applicability of available genetic information in the Basque population are scarce. In total, 835 CRC cases and 940 controls from the Basque population were genotyped and genome-wide association studies were carried out. Mendelian Randomization analyses were used to discover the effect of modifiable risk factors and microbiota on CRC. In total, 25 polygenic risk score models were evaluated to assess their performance in CRC risk calculation. Moreover, 492 inflammatory bowel disease cases were used to assess whether that genetic information would not confuse both conditions. Five suggestive (*p* < 5 × 10^−6^) *loci* were associated with CRC risk, where genes previously associated with CRC were located (e.g., *ABCA12*, *ATIC* or *ERBB4*). Moreover, the analyses of CRC locations detected additional genes consistent with the biology of CRC. The possible contribution of cholesterol, BMI, Firmicutes and Cyanobacteria to CRC risk was detected by Mendelian Randomization. Finally, although polygenic risk score models showed variable performance, the best model performed correctly regardless of the location and did not misclassify inflammatory bowel disease cases. Our results are consistent with CRC biology and genetic risk models and could be applied to assess CRC risk in the Basque population.

## 1. Introduction

In total, 10% of the cancers diagnosed in the world are colorectal cancers (CRC) and, in addition, CRC is the second cause of cancer death in developed countries [1,2]. The development of CRC can be sporadic or due to inflammatory processes [3]; the risk of CRC is influenced by the environment, genetics, and microbial composition [4,5]. Since CRC is a major public health issue, different strategies for its early detection and prognosis have been proposed and developed [6].

As mentioned, genetic factors are involved in CRC risk, or they can be associated with other risk factors related to CRC. As a consequence, their utility as biomarkers has been explored: their role in CRC risk has been studied by analyzing specific genetic variants [7,8,9], as well as, genome-wide association studies (GWAS) [10]. Moreover, the effect of genetic information on modifiable risk factors (e.g., lipids level) on CRC has been analyzed using Mendelian Randomization analyses [11,12], a method to estimate causal effects if specific assumptions are fulfilled. In addition, it has been detected that some genetic variants involved in the abundance of some microbial groups are related to CRC risk [13]. Finally, it has been proposed that polygenic risk scores (PRS) derived from different genetic studies are useful to predict the risk of CRC of one individual based on the carriership of risk genetic variants, among other factors [14,15].

Previously, 48 SNPs associated with CRC were analyzed in 230 CRC cases and 230 controls from the Basque population [16]. From those analyzed SNPs, only rs6687758 SNP was associated with CRC risk, and the application of those 48 SNPs as a model to predict PRS risk was successful [16]. Indeed, the Basque population has a particular genetic history compared to the rest of the European population, since the migrations associated with the Steppe pastoralism had less effect on that population, therefore, genetic variants from populations that lived in Europe in the Neolithic [17] or Iron Age [18] could be higher. Previously, a genetic study of this cohort showed that it was useful to study the effect of local genetic variants on the risk and ability to predict the risk of complex diseases [19]. In addition, according to the data available from the Basque Statistic Institute (https://en.eustat.eus, accessed: 1 August 2022), between 2016 and 2019, in the Basque Autonomous Community (Northern Spain) CRC caused 8356 hospitalizations (on average, 95.58 hospitalizations per 100.000 habitants per year), while in the rest of Spain there were 101.12 hospitalizations per 100.000 habitants per year (between 2016–2019, according with Instituto Nacional de Estadística, https://www.ine.es, accessed: 1 August 2022), and in Europe, there were 123.45 hospitalizations per 100.000 habitants per year (between 2016–2019, according to Eurostat, https://ec.europa.eu/eurostat, accessed: 1 August 2022).

In the present study, we analyze a larger Basque cohort (835 cases and 940 controls) to detect the risk factors for CRC that can be explained or inferred from the genetic component of CRC using genome-wide association studies and Mendelian Randomization to assess the applicability of existing CRC PRS models on this population.

## 2. Materials and Methods

### 2.1. Recruitment

CRC cases were diagnosed using standard criteria and the samples used in this study were obtained in the standard clinical practice, after informed consent, in Hospital Universitario Donostia (San Sebastian, Spain). The samples of non-CRC controls were obtained through the Basque Biobank; the samples were sourced from healthy blood donors (the age range to be eligible to be a blood donor is 18–65). The information of those blood donors is anonymized and only information about sex and age is made available. In total, 869 cases were recruited, and 987 controls were used.

The present study was approved by the Local Ethics Committee (Comité de Ética de la Investigación con medicamentos de Euskadi, code: PI+CES-BIOEF 2017-10).

### 2.2. Genotyping and Imputation

Illumina Global Screening Array was used to genotype the DNA samples of the individuals analyzed in this work. For this, Illumina iScan high-throughput screening system was used in the Institute of Clinical Molecular Biology (Kiel, Germany). Raw intensities were transformed to alleles using the GenCall algorithm available in Illumina GenomeStudio software.

Then, the called genotypes and samples were filtered using the following criteria: samples with ≥5% missing rates; markers with non-called alleles; markers with missing call rates > 0.05; related samples (PI-HAT > 0.1875); samples whose genotyped sex could not be determined; samples with high heterozygosity rate (more than 3 times SD from the mean) were excluded. In addition, only autosomal SNPs were kept; markers with Hardy–Weinberg equilibrium *p* < 1 × 10^−5^; markers whose P of difference in missingness between cases and control was <1 × 10^−5^; samples that were outliers, identified using principal component analysis (deviation of more than 6 times interquartile range), using FlashPCA (v2.0) [20], were removed.

Additional SNPs were imputed using the Sanger Imputation service. Release 1.1 of the Haplotype Reference Consortium was used as a reference panel, and the EAGLE2+PBWT pipeline was used to carry out the imputation [21,22,23]. Once imputed, markers with INFO score < 0.80, MAF < 0.01 and non-biallelic markers were removed.

After genotyping, quality control and imputation, 5,399,981 SNPs from 1775 individuals (835 cases and 940 controls) were kept.

### 2.3. Genetic Analyses

#### 2.3.1. Admixture Analysis

Genotyped SNPs were pruned using Plink (v1.90) [24] and SNPs from regions with high linkage disequilibrium were removed. Admixture (v1.3) [25] was used to analyze the admixture of the samples of our cohort, with settings K between 1 and 10, and using the results with the lowest cross-validation value.

#### 2.3.2. Genome-Wide Association Study

GWAS analyses of CRC cases and non-CRC controls were performed using logistic regression implemented in Plink [24], adjusting by sex, age and the first 4 principal components. In addition, GWAS of right colon cancer, left colon cancer, and rectal cancer vs non-CRC controls, as well as right colon cancer vs left colon cancer, and colon cancer vs rectal cancer were carried out using logistic regression implemented in Plink, and adjusting by sex, age and first 4 principal components.

To compare our results with SNPs previously associated with CRC, SNPs associated with the “Colorectal cancer” term (EFO_0005842) and studied in populations of European origin were retrieved from GWAS Catalog [26]. In total, 209 SNP from 34 studies were retrieved.

Moreover, CRC patients were compared to 492 inflammatory bowel disease patients without CRC [19] to find genetic differences in our cohort. To perform that analysis, a logistic regression implemented in Plink, adjusting by sex, age and first 4 principal components, was used. In addition, a comparison of CRC patients against the mentioned inflammatory bowel disease patients plus controls was carried out.

#### 2.3.3. Mendelian Randomization Analyses

For carrying out Mendelian Randomization (MR) analyses TwoSampleMR (v0.5.6) [27] and gsmr (v1.0.9) [28] packages from R language (v4.0.5) were used [29], as we have used previously to study the effect of modifiable risk factors in CRC risk [13].

First, we selected the modifiable risk factors based on a previous work [12] which analyzed modifiable risk factors using Mendelian Randomization that affects CRC (BMI, cholesterol, triglycerides, selenium, iron, vitamin B12, metabolism, body fat percentage, waist circumference, IL6 receptor and height). Then, we retrieved the instruments available in MRC-IEU (https://gwas.mrcieu.ac.uk, accessed: 14 February 2022) of those traits through TwoSampleMR [27]. In addition, to analyze the effect of the microbiota in CRC cancer, we retrieved instruments of bacterial phyla which are available from MiBioGen consortium data [30]. 

Then, the analysis was carried out if 10 or more instruments were available, and HEIDI outlier analysis was used to discard heterogenous instruments. The strength of the instruments was measured by the F-statistic: F = R^2^(N – K − 1)/K(1 − R^2^), where R^2^ is the variance explained by genetic variance, N is the sample size, and K is the number of instruments [31]. In addition, I^2^ was calculated using TwoSampleMR R Package.

The MR analyses were carried out using Inverse Variance Weighted, Weighted Median and MR Egger methods. In addition, the heterogeneity Q test and pleiotropy test available in TwoSampleMR R Package were used as sensitivity tests. The analysis was applied to all CRC cases, as well as, right colon cancer, left colon cancer and rectal cancer analyses.

#### 2.3.4. Polygenic Risk Scores

Polygenic risk scores (PRS) were retrieved from PGS Catalog [32]. 29 scores available in the “Colorectal cancer” term (EFO_0005842) derived using cohorts with >90% samples of European ancestry and whose assembly version was known were used for the PRS analysis [33,34,35,36,37,38,39,40,41]. From those 29 panels, our cohort had available SNPs to apply in 25 of them. In addition, the PRS used previously in the Basque population was tested [16]. The weights of the SNPs present in our data were applied in our cohort using Plink [24]. The performance of the PRS was measured by comparing the PRS score distribution of CRC cases and non-CRC controls using a T-test using R language [29]; the effect size of the T-test was calculated using Cohen’s d through the package rstatix (https://CRAN.R-project.org/package=rstatix, accessed: 28 April 2022) of R language, the area under de curve, sensitivity and specificity was calculated using pROC package of R language. The 95% of confidence interval of the area under the curve was calculated using that package and the DeLong method.

In addition, CRC PRS were applied in 492 patients with inflammatory bowel disease without CRC [19] to measure the ability to distinguish both conditions.

Additional statistical analyses and graphics were done using R language [29].

## 3. Results

In this study, we have analyzed 835 CRC cases and 940 population-based controls (Table 1). In the cases and the controls, around two-thirds of the individuals were males (63.47% and 67.13%, respectively), and cases were older (average age, 73.54) than the controls (average age, 41.53). The majority of the CRC patients were in stages II and III (37.61% and 26.71%, respectively), with located tumors in the rectum (28.14%) and left colon (26.23%) (Table 1).

The individuals with modern European ancestry overlapped with the Iberian population of 1000 Genomes data, while the ancient European ancestry was distanced from European populations (Supplementary Figure S1A). In addition, the PC1 of the principal component analysis of the samples was determined by the ancestry component of our cohort (Supplementary Figure S1B).

### 3.1. Genome-Wide Association Studies

The genome-wide association study of all CRC cases showed five suggestive (*p* < 5 × 10^−6^) signals (Table 2). The most significant SNP was rs77317240, located in chromosome 2 and upstream of *ABCA12* and *ATIC* genes (*p* = 5.8 × 10^−7^; OR = 6.4; CI 95%, 3.1–13.2). Other suggestive SNPs were located in *ERBB4* and *MAGI2* genes, and downstream of the *IL15* gene (Table 2). 

When cancer locations were analyzed separately different signals were detected (Table 2): 16 in right colon cancer (the most significant signal was located in the *NTF3* gene), 7 in left colon cancer (the most significant signal was located in the *ABCC12* gene), and 10 in rectal cancer (the most significant signal was located in *BRD7* gene). When locations were compared (Table 2), 2 signals were detected when comparing left and right colon cancers (the most significant genetic variant was located in the *FERMT2* gene) and 3 when comparing rectal vs colon cancers (the most significant genetic variant was located in *CNTNAP2* gene). 

Among the SNPs previously associated with CRC (Supplementary Table S1), 16 SNPs (7.65% of SNPs previously associated) showed nominal association in our cohort. When those SNPs were analyzed by the location of cancer, 9 (4.31%) were nominally significant in right colon cancer, 12 (5.74%) in left colon cancer (including rs6687758, an SNP previously associated with CRC in the Basque population) and 12 (5.74%) in rectal cancer. Among the 31 SNPs previously associated with CRC in more than one study (Supplementary Table S1), 5 SNPs (16.13%) showed nominal association in CRC; 3 (9.68%) in right colon cancer; 3 (9.68%) in left colon cancer and 1 (3.23%) in rectal cancer.

Regarding the comparison with inflammatory bowel disease (Table 3), 11 genomic regions had suggestive different frequencies. Among them, the signal located upstream of the *ATP8B4* gene (rs541295) reached a genome-wide significant *p*-value (*p* = 1.8 × 10^−8^). When colorectal cancer was compared with the pool of controls and inflammatory bowel disease (Table 3), the most significant signal in CRC vs controls (upstream of the *ABCA12* and *ATIC* genes) was detected. In addition, 4 of the signals detected when CRC was compared with inflammatory bowel disease patients were suggestive: in the HLA region, in the *DLGAP2* gene, downstream of the *PTCHD3* gene and upstream of the *ATP8B4* gene.

### 3.2. Mendelian Randomization

Mendelian Randomization analyses were carried out to analyze the effect of modifiable risk factors and the abundance of bacterial phyla on CRC risk. The instruments used seemed appropriate (Supplementary Table S2), although the modifiable risk factors were stronger than bacterial phyla (F-statistic between 55.82–211.35 in the former, 18.73–20.28 in the latter).

**Figure 1:**
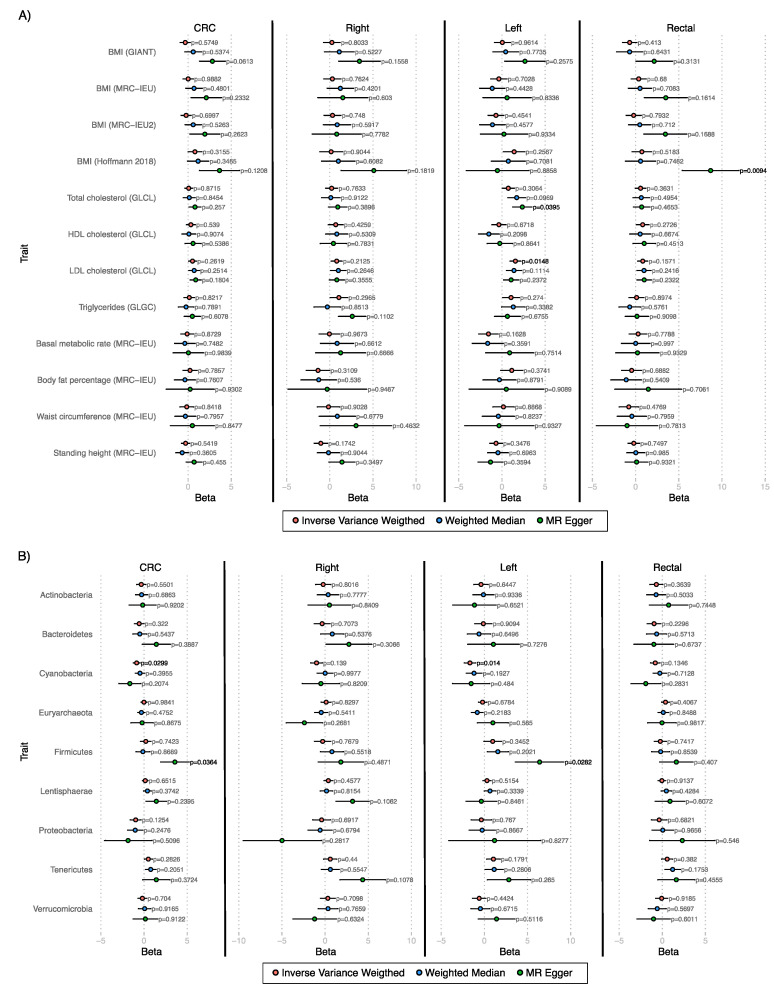
Mendelian Randomization results. The beta value and standard error are depicted for each method used. (**A**) Modifiable risk factors. (**B**) Bacterial phyla.

When analyzing the effect of modifiable risk factors on CRC, there were no significant results (Figure 1A, Supplementary Table S3). However, when the locations of CRC were separately analyzed, the MR Egger method showed the effect of total cholesterol (beta = 2.4 ± 1.1; *p* = 0.0395) on left-sided colon cancer risk, and the effect of BMI (beta = 8.7 ± 3.3; *p* = 0.0094) in rectal cancer risk. In the latter, pleiotropic effects were detected (*p* = 0.0112, Supplementary Table S3). In addition, Inverse Variance Weighted method showed the effect of LDL cholesterol (beta = 1.56 ± 0.64; *p* = 0.0148) on left-sided colon cancer risk.

In the case of bacterial phyla (Figure 1B, Supplementary Table S4), according to MR Egger method, Firmicutes phylum showed a significant effect on CRC and left colon cancer (beta=3.6 ± 1.7; *p* = 0.0364; beta = 6.4 ± 2.8; *p* = 0.0282, respectively), although pleiotropy was detected in both cases (*p* = 0.0347; *p* = 0.0456, respectively, Supplementary Table S4), as well as, heterogeneity in the used instruments (Q-test *p* = 0.0336 and *p* = 0.0107, respectively, Supplementary Table S4). In the case of Inverse Variance Weighted, there was an inverse effect of Cyanobacteria abundance on CRC risk and left colon cancer risk (beta = −0.86 ± 0.39; *p* = 0.0299; beta = −1.66 ± 0.68; *p* = 0.014, respectively).

### 3.3. Polygenic Risk Scores

**Figure 2:**
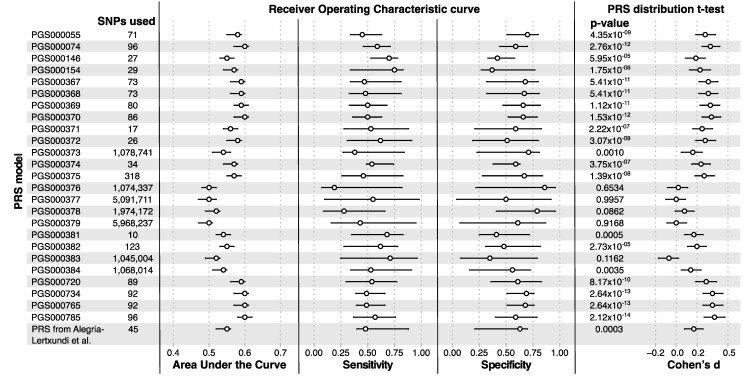
Performance of different Polygenic Risk Score sets. For each parameter, the value and 95% of confidence interval are depicted. Cohen’s d, the effect size of the T-test and 95% of the confidence interval, <0.2 negligible effects, 0.2–0.5 small effect, 0.5–0.8 moderate effect, >0.8 large effects.

**Figure 3:**
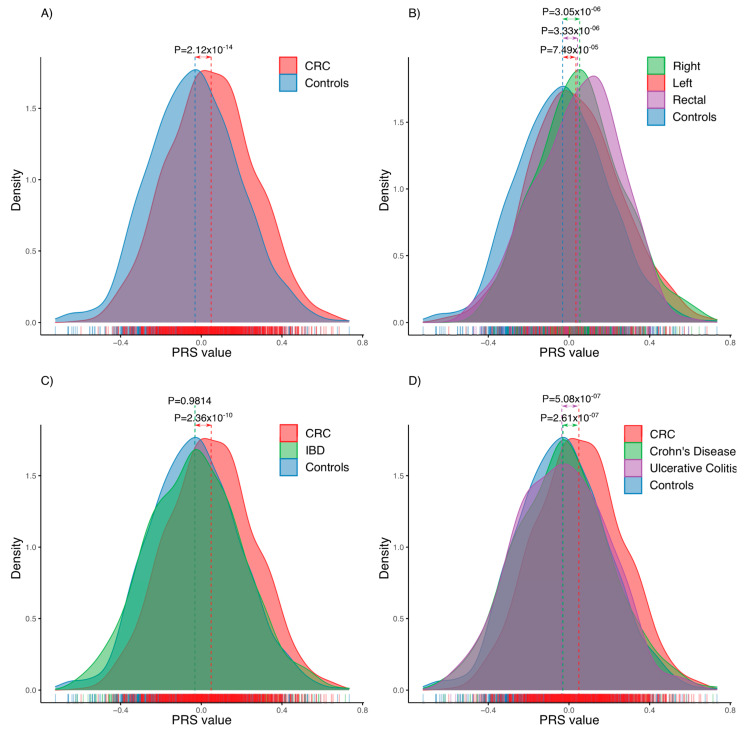
Distribution of polygenic risk score based on PGS000785 set. P, the *p*-value of the T-test. (**A**) Colorectal cancer. (**B**) According to the location. (**C**) Comparison with patients with irritable bowel disease. (**D**) Comparison with patients of main types of irritable bowel disease.

Polygenic risk scores for our cohort were built using 25 different models available in PGS Catalog for CRC. From all of them (Figure 2 and Figure 3A), PGS000785 showed the best discrimination between the PRS values for cases and controls (T-test *p* = 2.12 × 10^−14^; small effect according to Cohen’s d), as well as, the best AUC value (0.6, CI 95% 0.58–0.62); followed by PGS000734 and PGS000765 (both *p* = 2.64 × 10^−13^; small effect according to Cohen’s d; AUC of 0.6, CI 95% 0.57–0.61). In addition, the PRS used previously in a Basque cohort showed lower significance (*p* = 0.0003; negligible effect according to Cohen’s d) and AUC value (0.55, CI 95% 0.52–0.56).

The PGS000785 PRS model had a good performance regardless of the location of CRC (Figure 3B): the distribution of the PRS score was significantly higher in right colon cancer (*p* = 3.05 × 10^−6^), left colon cancer (*p* = 7.49 × 10^−6^) and rectal cancer (*p* = 3.33 × 10^−6^) compared to controls, while there were no significant differences comparing locations. In addition, that model was able to differentiate inflammatory bowel disease patients from colorectal cancer patients (*p* = 2.36 × 10^−10^, Figure 3C), regardless of the type of inflammatory bowel disease (Crohn’s Disease, *p* = 2.61 × 10^−7^; Ulcerative colitis, *p* = 5.08 × 10^−7^; Figure 3D).

## 4. Discussion

The development of colorectal cancer (CRC) is influenced by environmental factors [4], microbiome composition [5] and genetic factors. In this work, we have analyzed the contribution of the genetic component to CRC risk in the Basque population, a population with a particular genetic history. That particular genetic history was reflected in the principal component analysis and, as it was done before [19], adjusting for PCs is enough to avoid artifacts due to the presence of two ancestries in the population.

Previously, selected SNPs were analyzed in CRC in the Basque population [16] and, in this study, we have used a GWAS approach and increased the sample size. In that previous work, the SNP rs6687758 was nominally significant [16] and we have been able to detect the nominal significance of that SNP in left colon cancer, as well as more genetic variants. We are aware that the sample size affected the results we obtained, and, for example, few previously associated SNPs with CRC were detected in our study. However, we were able to find nominally significant results for the SNPs detected in more than one study. In addition, the majority of SNPs detected in previous studies were not detected in other studies. Thus, the genetic risk of CRC could be partially due to local variation, therefore, it seems appropriate for the genetic analysis of CRC in new populations.

The most significant signal in CRC, although it was not genome-wide significant, was located between *ABCA12* and *ATIC* genes. It has been reported that the expression of *ABCA12* is upregulated in CRC [42,43], its expression is higher in the colon than in the rectum [43], and its expression is higher in colorectal adenoma than in hyperplastic polyp [44]. In the case of the *ATIC* gene, it has been proposed that its expression could be a prognostic marker for colon adenocarcinoma [45]; its presence in small extracellular vesicles in serum is useful to differentiate early colorectal neoplasia from advanced colorectal neoplasia [46].

Another suggestive signal was located on the *ERBB4* gene. In cell culture and mice, it has been observed that *ERBB4* expression and signaling can prevent apoptosis of the cells in an inflammatory environment [47], therefore, its chronic overexpression could contribute to the appearance of tumors, since apoptosis of colonic cells is inhibited [48]. In humans, it has been reported the overexpression of *ERBB4* in CRC and that tumors with high levels of this receptor could have enhanced cell survival [49]. In addition, it has been suggested that the expression of *ERBB4* is associated with unfavorable clinical outcomes in CRC [50] and that it could be a marker of a higher risk of recurrence [51]. Additionally, it has been reported that *ERRB4* expression is positively associated with lymph node metastasis [50]; that *ERBB4* could play a relevant role in a gene network associated with progression from colon adenocarcinoma to liver metastases [52], and that *ERBB4* could be part of a pathway that enhances the invasion of CRC cells [53].

Additional suggestive signals were located in the *MAGI2* gene and downstream of the *IL15* gene. The SNP rs34931968 detected in our cohort is located in the *MAGI2* gene, upstream of a lncRNA that is next to *MAGI2* (called *MAGI2-AS3*), a lncRNA that has been involved in CRC [54,55,56]. In addition, the SNP rs34931968 is in linkage disequilibrium with an SNP (rs7783388) involved in CRC throughout changes in *MAGI2-AS3* expression [56]. In the case of *IL15*, its expression has been associated with the outcome of CRC [57].

When the locations of the tumors were analyzed separately, other possible relevant genes were detected. In right colon cancer, the most significant signal was located in *NTF3*, a gene implicated in unfavorable prognosis in hepatocellular carcinoma [58,59]; in left colon cancer *ABCC12* gene, another ATP-binding cassette as the previously discussed *ABCA12*; in rectal cancer *BRD7* gene, a possible oncogene involved in CRC progression [60]. In addition, in rectal cancer the SNP rs13403794 was detected, an SNP located upstream of *ADAM17*, which is a gene that is part of the signaling pathway involved in colorectal cancer progression and chemoresistance [61]. When locations were compared, additional genes were detected: *FERMT2*, whose overexpression in CRC has been detected and associated with cell growth [62]; *CNTNAP2*, a gene that has not been associated with CRC. It has been observed that the genetic mechanisms behind CRC could be different depending on its location [63] and the differences in the genetic variants detected in our study are consistent with that suggestion.

On the whole, considering the biological role of some of the genes where the suggestive genetic variants were located, those genetic variants could be markers of the progression of CRC, at least in the Basque population, although follow-up analyses are needed to confirm their potential utility as markers.

Various modifiable risk factors have been observed to affect CRC risk [11,12,64], but we were not able to find those effects when all CRC patients were analyzed. However, when each location was analyzed, the effect of genetic risk to higher cholesterol levels (general levels or LDL) on left colon cancer and higher BMI on rectal cancer were detected, as has been suggested previously for CRC [11,12,13,64,65]. Although we tried to replicate the results obtained using Mendelian Randomization in previous works [12,13] and the traits and instruments used seem appropriate to replicate them, the results we obtained were limited or were detected only by one method. It could be possible that the size and characteristics of our cohort and GWAS analyses complicate the finding of clear causalities, since the traits we used to have strong instruments to avoid the biases of our cohort.

The genetic signature of the abundance of Firmicutes was associated with a higher risk of CRC and left colon cancer in our cohort, although the results should be taken with caution since heterogeneity was detected. In addition, that association had a pleiotropic effect, that is, rather than the presence of Firmicutes affecting the risk of CRC (cause and effect), there is a shared genetic component that affects both (common biologic mechanism). It has been described the importance of the microbiota in CRC risk and development [66,67], the differences in its composition between left and right colon cancer [68,69,70] and shared genetic variants in CRC risk and the abundance of Firmicutes [13]. Although the connection we have detected between CRC and Firmicutes is based only on their shared genetic variants, it has been observed that the involvement of Firmicutes in CRC risk was variable [68,69,70,71]: some genera of Firmicutes were enriched in CRC while others were depleted. In the case of Cyanobacteria, a higher abundance of that phylum has been observed in colorectal adenomas [72], and in animal models, it has been observed a higher abundance of Cyanobacteria when oxaliplatin is administered [73]. Therefore, follow-up analyses of Firmicutes and Cyanobacteria as a marker of CRC risk in the Basque cohort are needed. Although the involvement of Firmicutes and Cyanobacteria in CRC seems biologically possible, their connection through Mendelian Randomization in our work seems weak, since they have been detected only by one method. In addition, although the study of the effect of host genetics on microbial abundance has been a valuable resource [30], it could be possible that the available instruments are not still appropriate to carry out Mendelian Randomization analyses, at least in our cohort.

Finally, polygenic risk scores (PRS) have been proposed as a tool for risk prediction in colorectal cancer [15]. We applied several publicly available PRS models, and their performance was variable. The best model was built using different sources available in GWAS Catalog and the interplay between genetic risk and modifiable risk factors [37]. In the case of CRC, that work suggested that PRS was the primary determinant of risk stratification in their application of the PRS model in UK Biobank data [37]. Although our cohort has a slightly different genetic background, since there is a higher genetic component of ancient European ancestry, the application of the PRS was able to differentiate CRC cases from controls, regardless of the location of the tumors. Since the AUC was low and the effect small, additional genetic or non-genetic risk factors should be incorporated to build a model for better discrimination. In addition, this PRS did not confuse CRC and inflammatory bowel disease or its main types in our cohort, suggesting that when there are overlapping symptoms, the use of that PRS would not misclassify an IBD patient as a CRC patient. In addition, we found genetic variants that could be used to discriminate between CRC and inflammatory bowel disease in our cohort, although follow-up analyses are needed. Regarding the PRS previously used in Basques [16], the performance in our data was not as good as the best model, but the controls showed lower PRS than CRC cases (*p* = 0.003), similar to the previous analysis of Basques (*p* = 0.002 for the unweighted values, *p* = 0.036 for weighted values) [16]. Therefore, the incorporation of a different set of SNPs for the development of more precise PRS models is still necessary, and the performance of PRS models should be investigated in additional samples of this population.

Considering the results obtained in the different analyses we have carried out since the results are quite consistent with previous results, genetic CRC risk in the Basque population seems to be similar to other European populations. The suggestive signals from the GWAS were consistent with CRC biology, although in some variants the frequency in the Basque population was quite different. Mendelian Randomization analyses did not find clear causal relationships, although the traits used were reported to affect CRC risk in other cohorts, therefore, follow-up studies are needed to assess if our results are due to methodological constraints or differences in the specific mechanisms. Finally, the application of polygenic risk scores based on European populations seemed a feasible approach to capture the CRC risk in the Basque population, although they can be improved. Thus, as happened in inflammatory bowel disease [19], the genetic architecture of CRC risk in the Basque population is similar to other European populations but local genetic variation shapes the risk.

## 5. Conclusions

In conclusion, we have analyzed the genetic component of the risk of CRC in the Basque population. Although the sample size was limited and there were constraints in the analyses due to the cohort used, we detected genetic factors whose involvement in the risk of CRC is consistent with the biological mechanisms of CRC, and we identified plausible genetic markers and an appropriate polygenic risk score model to assess the genetic contribution to CRC risk in this population. In the future, those genetic factors and the polygenic risk score model should be validated in follow-up studies.

## Figures and Tables

**Table 1:** Demographics of the participants.

	Cases	Controls
N	835	940
Male (%)	530 (63.47%)	631 (67.13%)
Female (%)	305 (36.53%)	309 (32.87%)
Age (SE)	73.54 (11.38)	41.53 (11.79)
Stage		
0	37 (4.43%)	
I	130 (15.57%)	
II	314 (37.61%)	
III	223 (26.71%)	
IV	105 (12.57%)	
Undetermined	26 (3.11%)	
Location		
Right	170 (20.36%)	
Left	219 (26.23%)	
Rectal	235 (28.14%)	
Unspecific	211 (25.27%)	

**Table 2:** Suggestive signals (*p* < 5 × 10^−6^) detected in colorectal cancer and the locations. Gene, gene where is located the SNP or nearest gene 100kb upstream or downstream from the SNP. OR, odds ratio. CI 95%, confidence interval of 95% of the odds ratio. Freq, frequency of A1 in Basque cohort. Freq EUR, frequency of A1 in European populations of 1 KG.

Lead SNP	Position	Gene	A1	A2	OR (CI 95%)	*p*-Value	Freq	Freq EUR
Colorectal cancer vs. controls								
rs79374732	2:212815957	*ERBB4*	T	C	8.5 (3.4–21.0)	4.5 × 10^−6^	0.032	0.022
rs77317240	2:216091445	Upstream of *ABCA12* and *ATIC*	T	C	6.4 (3.1–13.2)	5.8 × 10^−7^	0.039	0.024
rs116443146	4:142699393	Downstream of *IL15*	G	A	16.3 (5.0–53.8)	4.4 × 10^−6^	0.013	0.02
rs34931968	7:79055118	*MAGI2*	T	G	29.7 (7.1–124.3)	3.4 × 10^−6^	0.011	0.01
rs1693967	16:86289580	*LINC01081*	G	A	11.4 (4.1–32.1)	3.9 × 10^−6^	0.017	0.024
Right colon cancer vs. controls								
rs3004681	1:69054715	Downstream of *DEPDC1*	T	G	11.8 (4.3–32.7)	2.0 × 10^−6^	0.062	0.073
rs77445470	1:226800066	Downstream of *STUM* and *ITPKB*	G	C	18.5 (5.3–64.5)	4.8 × 10^−6^	0.044	0.055
rs76653793	4:47962934	*CNGA1*, *LOC101927157*	G	T	21.7 (6.4–73.8)	7.9 × 10^−7^	0.028	0.036
rs142444738	4:106095747	*TET2, TET2-AS1*	A	G	51.1 (9.6–270.9)	3.8 × 10^−6^	0.011	0.005
rs4696337	4:153602674	*TMEM154, LOC105377495*	A	C	35.8 (8.2–156.2)	2.0 × 10^−6^	0.023	0.023
rs139432545	4:174624195		G	A	48.4 (9.6–244.9)	2.7 × 10^−6^	0.012	0.022
rs13211079	6:36977349	*FGD2*	G	C	43.9 (9.2–210.2)	2.2 × 10^−6^	0.019	0.012
rs190591066	7:89988294	*GTPBP10*	A	G	40.6 (8.8–186.4)	1.9 × 10^−6^	0.017	0.011
rs75772232	8:83689525		T	C	15.8 (4.9–51.2)	4.3 × 10^−6^	0.039	0.045
rs118025264	9:119407781	*ASTN2*, *LOC105376240*	T	C	25.7 (6.4–102.7)	4.3 × 10^−6^	0.026	0.022
rs16933489	12:5572210	*NTF3*	T	C	34.9 (9.1–133.3)	2.0 × 10^−7^	0.02	0.044
rs78263620	18:72995680	*TSHZ1*	T	C	43.6 (9.2–207.9)	2.2 × 10^−6^	0.011	0.019
rs148452202	19:2527577	*GNG7*	A	G	34.6 (8.3–144.8)	1.2 × 10^−6^	0.022	0.01
rs35914129	19:48115566	*BICRA*	T	G	56.2 (11.2–283.0)	1.0 × 10^−6^	0.013	0.009
rs28495197	22:36050632	*APOL6*	T	C	39.9 (9.1–174.2)	9.4 × 10^−7^	0.023	0.017
rs117820381	22:40738486	Downstream of *TNRC6B*, upstream of *ADSL*	A	G	37.0 (8.4–163.1)	1.8 × 10^−6^	0.013	0.028
Left colon cancer vs. controls								
rs112033525	2:23176856		T	G	39.4 (8.2–189.6)	4.5 × 10^−6^	0.017	0.015
rs139367040	2:173950614	*MAP3K20*	T	C	33.0 (7.7–142.5)	2.8 × 10^−6^	0.019	0.014
rs72774468	9:137697318	*COL5A1*	C	T	15.1 (5.0–45.3)	1.3 × 10^−6^	0.035	0.051
rs114144417	16:48116976	*ABCC12*	T	C	149.8 (20.2–1112.0)	9.7 × 10^−7^	0.01	0.008
rs17721600	17:27268513	*PHF12*, *LOC101927018*	A	G	25.9 (6.9–97.7)	1.6 × 10^−6^	0.037	0.053
rs140107269	18:1828990		T	C	26.8 (6.6–109.2)	4.4 × 10^−6^	0.023	0.027
rs62093285	18:49252189		A	G	12.8 (4.3–38.4)	4.9 × 10^−6^	0.044	0.035
Rectal cancer vs. controls								
rs78144988	1:102199388	*LINC01709*	C	T	54.9 (11.2–268.4)	7.6 × 10^−7^	0.013	0.018
rs13403794	2:9785060	Upstream of *YWHAQ* and *ADAM17*	C	T	65.5 (12.0–355.9)	1.3 × 10^−6^	0.012	0.021
rs354856	2:142433670	*LRP1B*, *LOC107985779*	C	T	17.4 (5.5–55.0)	1.1 × 10^−6^	0.027	0.062
rs116443146	4:142699393	Downstream of *IL15*	G	A	40.3 (9.2–176.9)	9.7 × 10^−7^	0.013	0.02
rs72909399	6:86581045		T	G	74.7 (13.5–414.7)	8.1 × 10^−7^	0.014	0.03
rs71516114	8:784674	*DLGAP2*	C	T	5.2 (2.6–10.4)	2.7 × 10^−6^	0.111	0.112
rs61848097	10:50134508	*WDFY4*, *LRRC18*	G	A	8.6 (3.5–21.0)	2.9 × 10^−6^	0.073	0.089
rs77470802	14:27547598	*LOC105370420*	G	T	12.4 (4.2–36.5)	4.6 × 10^−6^	0.027	0.033
rs76799782	14:91624544	*DGLUCY*	A	G	18.9 (5.4–65.4)	3.8 × 10^−6^	0.029	0.039
rs141553824	16:50380386	*BRD7*	C	T	45.8 (10.4–202.4)	4.5 × 10^−7^	0.017	0.05
Left colon cancer vs. right colon cancer								
rs4655303	1:213834643	*LOC105372912*	T	A	2.2 (1.6–3.0)	3.6 × 10^−6^	0.43	0.377
rs62005704	14:53465150	Downstream of *DDHD1*, upstream of *FERMT2*	A	G	0.4 (0.3–0.6)	9.8 × 10^−7^	0.464	0.503
Rectal cancer vs. colon cancer								
rs73171906	7:147986529	*CNTNAP2*	T	C	2.2 (1.6–2.9)	6.4 × 10^−7^	0.23	0.154
rs9773025	8:6674458	*XKR5*	G	A	0.5 (0.3–0.6)	1.5 × 10^−6^	0.414	0.468
rs79619562	21:38742422	*DYRK1A*	C	T	2.7 (1.8–4.1)	1.8 × 10^−6^	0.1	0.093

**Table 3:** Suggestive signals (*p* < 5 × 10^−6^) detected in the comparison of colorectal cancer and inflammatory bowel disease. Gene, gene where is located the SNP or nearest gene 100kb upstream or downstream from the SNP. OR, odds ratio. CI 95%, confidence interval of 95% of the odds ratio. Freq, frequency of A1 in Basque cohort. Freq EUR, frequency of A1 in European populations of 1 KG.

Lead SNP	Position	Gene	A1	A2	OR (CI 95%)	*p*-Value	Freq	Freq EUR
Colorectal cancer vs inflammatory bowel disease								
rs35493687	1:41285292	*KCNQ4*	A	C	0.4 (0.3–0.6)	4.2 × 10^−6^	0.122	0.147
rs76845271	2:73665817	*ALMS1*	T	G	0.3 (0.2–0.5)	2.9 × 10^−6^	0.043	0.048
rs6738805	2:231083171	*SP110*	C	T	0.4 (0.3–0.6)	4.6 × 10^−7^	0.135	0.128
rs10007784	4:81977690	*BMP3*	C	T	0.5 (0.4–0.7)	1.8 × 10^−6^	0.228	0.222
rs181206673	5:25834969		C	G	0.3 (0.1–0.5)	4.1 × 10^−6^	0.039	0.0467
rs72840740	6:18745458		C	T	0.1 (0.0–0.2)	1.1 × 10^−6^	0.014	0.03
rs9271365	6:32586794	Downstream of *HLA-DRB1* and upstream of *HLA-DQA1*	G	T	1.8 (1.4–2.3)	2.2 × 10^−6^	0.353	0.388
rs951197	6:103210765		C	A	0.5 (0.4–0.7)	5.6 × 10^−7^	0.476	0.446
rs1875664	8:827824	*DLGAP2*	G	A	2.3 (1.6–3.3)	2.8 × 10^−6^	0.128	0.161
rs988874	10:27684660	Downstream of *PTCHD3*	A	T	0.5 (0.3–0.6)	1.6 × 10^−6^	0.174	0.157
rs541295	15:50056050	Upstream of *ATP8B4*	G	A	0.2 (0.1–0.4)	1.8 × 10^−8^	0.055	0.022
Colorectal cancer vs. controls + inflammatory bowel disease								
rs7550486	1:14777040	*KAZN*	C	T	0.6 (0.5–0.7)	1.3 × 10^−6^	0.498	0.475
rs115681984	2:216032071	Upstream of *ABCA12* and *ATIC*	T	C	4.2 (2.4–7.1)	2.6 × 10^−7^	0.034	0.026
rs72840741	6:18747455		G	A	0.1 (0.0–0.2)	1.8 × 10^−6^	0.014	0.03
rs5002178	6:32611590	*HLA-DQA1*	G	A	0.6 (0.5–0.7)	6.8 × 10^−7^	0.33	0.374
rs951197	6:103210765		C	A	0.6 (0.5–0.7)	2.4 × 10^−7^	0.484	0.446
rs1875664	8:827824	*DLGAP2*	G	A	2.2 (1.6–3.0)	3.24 × 10^−7^	0.124	0.161
rs988874	10:27684660	Downstream of *PTCHD3*	A	T	0.5 (0.3–0.6)	2.0 × 10^−6^	0.171	0.157
rs150840049	14:59165709	Downstream of *DACT1*	C	T	0.1 (0.1–0.3)	2.6 × 10^−6^	0.025	0.052
rs541295	15:50056050	Upstream of *ATP8B4*	G	A	0.2 (0.1–0.4)	5.3 × 10^−8^	0.045	0.022

## Data Availability

The data presented in this study are available on request from the authors. The data are not publicly available due to ethical reasons (genotype data cannot be shared).

## Supplementary Materials

The following supporting information can be downloaded at: https://www.mdpi.com/article/10.3390/cancers14174193/s1, Figure S1: PCA plots of analyzed Basque cohort; Table S1: Results of SNPs previously associated with CRC; Supplementary Table S2: Sensitivity analyses of used instruments in Mendelian Randomization analyses; Supplementary Table S3: Results of Mendelian Randomization analyses using modifiable risk factors as exposures; Supplementary Table S4: Results of Mendelian Randomization analyses using bacterial phyla as exposures.
