# Supplementary material for: Performance of the Use of Genetic Information to Assess the Risk of Colorectal Cancer in the Basque Population
Source: Cancers (Basel). 2022 Aug 29;14(17):4193. doi: 10.3390/cancers14174193 (PMC9454881; doi:10.3390/cancers14174193)
Supplement: Supplementary file 1 [file cancers-14-04193-s001.zip › cancers-1797985-supplementary.pdf]

Performance of the use of genetic information to assess the risk of colorectal cancer in the Basque population

Item	Page
**Supplementary Figure S1:** PCA plots of analysed Basque cohort. A) With 1000 Genome European populations. B) Alone.	2
**Supplementary Table S1**: Results of SNPs previously associated with CRC.	3
**Supplementary Table S2:** Sensitivity analyses of used instruments in Mendelian Randomization analyses.	9
**Supplementary Table S3**: Results of Mendelian Randomization analyses using modifiable risk factors as exposures.	10
**Supplementary Table S4**: Results of Mendelian Randomization analyses using bacterial phyla as exposures.	11

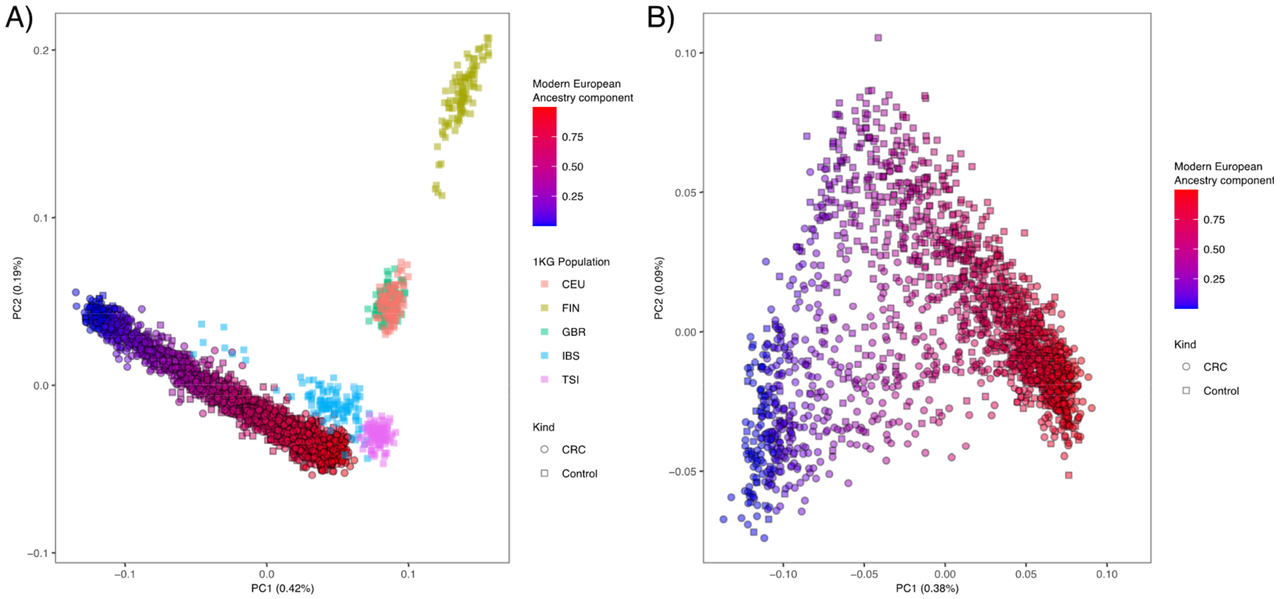

Supplementary Figure S1: PCA plots of analysed Basque cohort. A) With 1000 Genome European populations. B) Samples of analysed Basque cohort.

Supplementary Table S1: Results of SNPs previously associated with CRC. P, P-value; OR, Odds-ratio. Freq EUR, frequency of the minor allele in European populations of 1KG. Freq, frequency of the minor allele in Basque cohort.

				CRC			Right			Left			Rectal		
**SNP**	**Position**	**Freq EUR**	**P**	**OR**	**Freq**	**P**	**OR**	**Freq**	**P**	**OR**	**Freq**	**P**	**OR**	**Freq**	**Reference**
rs11803759	1:14665131	0.032	0.131	0.5	0.024	0.821	0.9	0.029	0.132	0.3	0.022	0.214	0.5	0.024	[1]
rs72647484	1:22587728	0.084	0.365	0.8	0.085	0.242	0.6	0.092	**0.015**	0.3	0.093	0.748	0.9	0.093	[2.3]
rs4949330	1:31449766	0.027	0.196	2.4	0.011	0.104	4.8	0.011	0.957	1.1	0.01	0.412	2.5	0.01	[1]
rs61776719	1:38461319	0.467	0.782	1.0	0.48	0.813	1.1	0.483	0.888	1.0	0.483	0.632	1.1	0.483	[3]
rs12143541	1:55247852	0.133	0.873	1.0	0.102	0.449	0.7	0.099	0.316	1.5	0.098	0.778	0.9	0.093	[3]
rs3753366	1:68195133	0.178	0.334	1.2	0.172	0.623	1.2	0.174	0.828	1.1	0.177	0.621	1.2	0.164	[1]
rs6668441	1:102242917	0.051	0.128	0.6	0.038	0.766	0.8	0.041	0.501	0.7	0.039	0.293	0.6	0.038	[1]
rs1912453	1:162821291	0.374	0.401	0.9	0.398	0.332	0.8	0.387	0.577	1.2	0.4	**0.047**	0.6	0.384	[4]
rs2027077	1:183073453	0.438	0.119	0.8	0.39	0.108	0.7	0.402	0.426	0.8	0.402	**0.045**	0.6	0.4	[5]
rs10911251	1:183081194	0.453	0.180	0.8	0.402	0.131	0.7	0.414	0.365	0.8	0.413	0.117	0.7	0.412	[4.6]
rs4381148	1:183803426	0.432	0.783	1.0	0.41	0.759	0.9	0.403	0.177	1.4	0.403	0.294	0.8	0.401	[5]
rs17626293	1:195651519	0.027	0.213	0.5	0.02	0.660	0.6	0.021	0.510	0.6	0.023	0.317	0.4	0.02	[1]
rs6691170	1:222045446	0.401	**0.049**	1.4	0.389	0.668	1.1	0.384	0.069	1.6	0.384	0.174	1.4	0.377	[7]
rs6658977	1:222049820	0.404	0.059	1.4	0.387	0.672	1.1	0.382	0.057	1.7	0.383	0.187	1.4	0.376	[3]
rs12140498	1:222098690	0.223	0.081	1.4	0.21	0.640	0.9	0.207	**0.008**	2.3	0.206	0.167	1.5	0.213	[5]
rs6687758	1:222164948	0.222	0.076	1.4	0.19	0.860	1.1	0.193	**0.003**	2.7	0.192	0.371	1.3	0.191	[6.7]
rs1202519	1:230947140	0.438	0.747	1.1	0.482	0.649	0.9	0.474	0.840	1.0	0.475	0.441	1.2	0.479	[1]
rs2184857	1:240081747	0.355	0.580	0.9	0.333	0.360	0.7	0.33	0.345	0.8	0.325	0.878	1.0	0.331	[1]
rs10495672	2:18494455	0.265	0.781	1.1	0.221	0.701	1.1	0.228	0.387	1.3	0.229	0.709	1.1	0.225	[1]
rs11692435	2:98275354	0.083	0.326	1.5	0.05	**0.039**	3.2	0.05	0.457	1.6	0.047	0.189	2.1	0.046	[3]
rs12999616	2:98324381	0.212	0.680	0.9	0.271	0.785	1.1	0.278	0.574	1.2	0.273	**0.049**	0.6	0.268	[1]
rs11123333	2:116909251	0.408	0.468	1.1	0.407	0.831	0.9	0.41	0.721	1.1	0.413	0.861	1.0	0.411	[1]
rs11903757	2:192587204	0.17	0.830	1.1	0.137	0.182	0.6	0.131	0.726	1.1	0.135	0.416	1.3	0.135	[4]
rs11893063	2:199601925	0.479	0.223	1.2	0.444	0.545	1.2	0.437	**0.025**	1.9	0.445	0.847	1.1	0.447	[3]
rs11884596	2:199612407	0.384	0.367	1.2	0.374	0.456	1.2	0.377	0.315	1.3	0.377	0.427	1.2	0.385	[3]
rs12612141	2:199871031	0.306	**0.025**	1.5	0.278	0.542	1.2	0.275	**0.024**	2.0	0.269	**0.009**	1.9	0.277	[5]
rs7593422	2:200131695	0.479	0.128	0.8	0.465	0.482	0.8	0.483	0.152	0.7	0.477	0.223	0.8	0.476	[3]
rs115481520	2:213257658	0.022	0.052	2.3	0.042	0.084	3.2	0.038	0.379	1.8	0.041	0.065	3.1	0.038	[8]
rs992157	2:219154781	0.416	0.226	0.8	0.472	0.249	0.7	0.468	0.379	0.8	0.475	0.120	0.7	0.463	[6.9]
rs13020391	2:219184436	0.337	0.307	0.8	0.391	0.368	0.8	0.39	0.878	1.0	0.398	**0.037**	0.6	0.386	[3]
rs7606512	2:235609674	0.034	**0.006**	2.9	0.044	**0.039**	3.7	0.043	0.207	2.7	0.04	0.062	2.9	0.041	[1]
rs57751578	3:24527581	0.101	0.830	1.1	0.097	0.620	0.8	0.097	0.212	1.6	0.102	0.861	0.9	0.097	[10]
rs139372065	3:28513403	0.017	0.075	2.5	0.028	0.563	2.0	0.023	0.063	4.6	0.026	**0.020**	4.9	0.027	[5]
rs35470271	3:40915239	0.158	**0.047**	1.9	0.102	0.664	1.2	0.102	**0.030**	2.7	0.103	0.271	1.7	0.099	[3]
rs35360328	3:40924962	0.156	**0.043**	1.9	0.106	0.857	1.1	0.107	**0.036**	2.6	0.109	0.357	1.5	0.104	[6.11]
rs8180040	3:47388947	0.399	0.718	1.1	0.408	0.624	1.1	0.408	0.612	1.1	0.416	0.598	0.9	0.405	[12]
rs17659990	3:50751149	0.043	0.855	1.1	0.032	0.593	0.6	0.027	0.361	1.7	0.031	0.709	0.8	0.029	[1]
rs353548	3:52269491	0.06	0.474	1.2	0.072	0.330	0.5	0.069	0.760	1.2	0.074	0.159	1.8	0.072	[13]
rs9831861	3:53088285	0.412	0.811	1.0	0.444	0.973	1.0	0.45	0.741	0.9	0.454	0.298	0.8	0.441	[3]
rs812481	3:66442435	0.441	0.579	1.1	0.436	0.300	1.3	0.459	0.316	1.3	0.451	0.555	0.9	0.442	[6.11]
rs11927424	3:88757831	0.361	0.580	1.1	0.327	0.646	0.9	0.335	0.826	0.9	0.331	0.533	0.9	0.326	[1]
rs12635946	3:112916918	0.399	0.948	1.0	0.428	0.673	1.1	0.433	0.966	1.0	0.431	0.859	1.0	0.434	[3]
rs12635946	3:112916919	0.399	0.856	1.0	0.428	0.970	1.0	0.433	0.995	1.0	0.431	0.906	1.0	0.434	[3]
rs16845107	3:113127991	0.062	0.173	0.6	0.051	0.403	0.6	0.053	0.132	0.4	0.053	0.103	0.4	0.056	[1]
rs57064586	3:133745796	0.099	0.430	0.8	0.073	**0.012**	0.2	0.075	0.731	0.8	0.075	0.586	1.3	0.075	[14]

rs35446936	3:169486508	0.242	0.772	1.1	0.189	0.482	1.3	0.196	0.668	1.1	0.194	0.894	1.0	0.183	[3]
rs10936599	3:169492101	0.242	0.761	1.1	0.19	0.476	1.3	0.196	0.660	1.1	0.194	0.902	1.0	0.183	[7]
rs3914272	3:171558536	0.163	0.185	1.3	0.181	0.285	1.5	0.179	0.079	1.8	0.179	0.501	1.2	0.178	[1]
rs13129679	4:2576102	0.013	**0.046**	0.3	0.022	0.605	0.6	0.023	0.479	0.6	0.025	0.069	0.2	0.023	[1]
rs16869961	4:20780315	0.301	0.178	1.3	0.32	0.055	1.8	0.312	0.113	1.6	0.309	0.715	1.1	0.305	[1]
rs6853410	4:82693536	0.251	**0.045**	0.7	0.26	0.713	0.9	0.267	0.087	0.6	0.264	0.553	0.9	0.264	[1]
rs13130787	4:94887031	0.428	0.333	1.2	0.497	0.584	1.1	0.499	0.257	0.8	0.487	0.900	1.0	0.496	[4]
rs17035289	4:106048291	0.19	0.374	0.8	0.163	0.671	0.9	0.16	0.558	1.2	0.169	0.811	0.9	0.166	[3]
rs75686861	4:145621328	0.077	0.720	1.1	0.075	0.231	1.9	0.076	0.559	1.3	0.076	0.715	0.8	0.075	[3]
rs35509282	4:163333405	0.110	0.534	1.2	0.087	0.905	1.1	0.086	0.377	0.6	0.081	0.285	1.6	0.085	[15]
rs77776598	5:1240998	0.053	0.414	1.4	0.04	0.087	2.8	0.044	0.470	0.6	0.04	0.605	1.4	0.042	[3]
rs2735940	5:1296486	0.489	0.582	1.1	0.492	0.843	0.9	0.486	0.719	0.9	0.484	0.054	1.5	0.494	[6]
rs17416314	5:31804351	0.082	0.520	0.8	0.075	0.878	0.9	0.079	0.919	1.1	0.082	0.150	0.5	0.077	[1]
rs1348612	5:40265904	0.255	0.308	1.2	0.315	0.088	1.6	0.309	0.407	1.2	0.302	0.307	1.3	0.311	[8]
rs17604996	5:126763087	0.035	0.171	0.5	0.014	0.885	0.9	0.014	0.108	0.2	0.015	0.139	0.2	0.014	[5]
rs4631227	5:132488440	0.308	0.251	1.2	0.289	0.688	1.1	0.29	0.710	1.1	0.285	0.245	1.4	0.289	[10]
rs639933	5:134467751	0.368	0.180	1.3	0.305	0.397	1.3	0.291	0.285	1.3	0.299	0.210	1.4	0.295	[3]
rs647161	5:134499092	0.34	0.290	1.2	0.35	0.409	1.3	0.347	0.240	1.4	0.346	0.564	0.9	0.351	[16]
rs845868	6:998813	0.182	0.872	1.0	0.167	0.108	1.7	0.171	0.810	1.1	0.168	0.291	0.7	0.17	[1]
rs742223	6:11705700	0.086	0.424	1.3	0.071	0.730	0.8	0.064	0.200	1.8	0.067	0.627	1.2	0.073	[1]
rs1362126	6:29691019	0.439	0.087	0.8	0.497	0.856	1.0	0.498	0.232	1.3	0.492	**0.044**	0.6	0.5	[1]
rs3131043	6:30758466	0.431	0.662	1.1	0.405	0.436	0.8	0.405	0.669	1.1	0.407	0.387	1.2	0.41	[3]
rs9271770	6:32594248	0.211	0.689	0.9	0.166	0.503	0.8	0.173	0.898	1.0	0.171	0.448	0.8	0.17	[3]
rs16878812	6:35569562	0.096	0.583	0.9	0.145	0.886	0.9	0.145	0.195	0.6	0.146	0.427	0.8	0.152	[3]
rs1321311	6:36622900	0.217	0.545	1.1	0.239	0.441	1.3	0.245	0.628	1.1	0.242	0.316	0.8	0.24	[17]
rs1321310	6:36623124	0.219	0.439	1.1	0.242	0.487	1.2	0.247	0.624	1.1	0.244	0.415	0.8	0.243	[3]
rs7742915	6:38147745	0.303	0.905	1.0	0.262	0.382	0.8	0.265	0.906	1.0	0.266	0.223	1.3	0.27	[1]
rs6933790	6:41672769	0.184	0.408	0.8	0.174	0.259	0.6	0.182	0.728	1.1	0.184	0.816	1.1	0.181	[3]
rs1155750	6:54730344	0.114	0.393	0.8	0.131	0.226	0.6	0.128	0.369	1.4	0.134	0.157	0.6	0.131	[1]
rs62404966	6:55712124	0.245	0.632	0.9	0.235	0.454	1.3	0.237	0.609	0.9	0.234	0.688	0.9	0.234	[3]
rs582962	6:69521978	0.302	0.912	1.0	0.255	0.068	0.5	0.259	0.096	0.6	0.256	0.101	1.6	0.265	[1]
rs72907251	6:87225368	0.062	0.275	1.6	0.045	0.053	3.3	0.045	0.798	0.8	0.04	0.447	1.6	0.043	[10]
rs2057314	6:117819357	0.485	0.346	1.2	0.49	0.342	1.3	0.469	0.775	1.1	0.477	**0.048**	1.6	0.484	[4]
rs4946260	6:117822993	0.484	0.346	1.2	0.49	0.342	1.3	0.469	0.775	1.1	0.477	**0.048**	1.6	0.484	[11]
rs9458376	6:162219854	0.016	0.366	0.5	0.016	0.525	0.4	0.017	0.556	1.7	0.016	0.566	0.6	0.015	[1]
rs3801081	7:47511161	0.3	0.966	1.0	0.342	0.431	0.8	0.345	0.927	1.0	0.342	0.924	1.0	0.341	[3]
rs17179503	7:81987289	0.043	0.482	1.5	0.021	0.696	1.5	0.022	0.963	1.0	0.022	0.426	1.8	0.022	[1]
rs6948772	7:155400895	0.32	0.339	1.2	0.342	0.308	1.3	0.343	0.226	1.4	0.346	0.740	0.9	0.343	[1]
rs1270639	7:157455459	0.122	0.640	0.9	0.084	0.927	1.0	0.089	0.830	1.1	0.09	0.165	0.5	0.084	[1]
rs7831492	8:41614503	0.405	0.691	0.9	0.403	0.633	0.9	0.408	0.816	1.1	0.408	0.811	0.9	0.406	[1]
rs72664998	8:69444614	0.023	0.707	0.8	0.026	0.355	1.9	0.024	0.681	0.7	0.023	0.636	1.4	0.024	[3.8]
rs112408346	8:75881879	0.019	0.684	0.8	0.021	0.981	1.0	0.018	0.701	1.4	0.02	0.936	0.9	0.02	[5]
rs3104964	8:96595736	0.4	0.051	1.4	0.427	0.405	1.2	0.436	0.741	1.1	0.429	0.120	1.4	0.43	[12]
rs1078186	8:101625479	0.417	0.538	0.9	0.417	0.991	1.0	0.414	0.723	1.1	0.425	0.732	1.1	0.412	[10]
rs16887197	8:116246667	0.083	0.957	1.0	0.063	0.178	0.5	0.058	0.666	0.8	0.059	0.726	1.2	0.064	[1]
rs2511653	8:117621592	0.193	0.987	1.0	0.175	0.860	1.1	0.182	0.335	1.4	0.178	0.403	1.3	0.179	[14]
rs16892766	8:117630683	0.090	**0.037**	1.9	0.053	**2.0E-04**	5.5	0.055	0.493	1.4	0.053	0.950	1.0	0.049	[6.11.18–20]
rs117079142	8:117790914	0.04	**0.021**	2.7	0.025	**3.3E-04**	8.7	0.025	0.542	0.5	0.021	0.152	2.5	0.021	[5]
rs10505477	8:128407443	0.479	**0.011**	0.7	0.454	**0.013**	0.5	0.459	0.189	0.7	0.465	0.504	0.9	0.47	[9.21]
rs6983267	8:128413305	0.499	**0.008**	0.6	0.442	**0.017**	0.5	0.45	0.107	0.7	0.453	0.310	0.8	0.459	[4.9.11.15.18.20.22]
rs7014346	8:128424792	0.335	0.054	1.4	0.368	0.226	1.4	0.354	0.130	1.5	0.358	0.967	1.0	0.35	[2.5.23]
rs17382698	8:129139206	0.132	0.817	1.1	0.168	0.883	0.9	0.163	0.202	1.6	0.167	0.640	1.2	0.164	[1]

rs2128382	8:130820039	0.167	0.975	1.0	0.169	0.626	1.2	0.177	0.746	1.1	0.17	0.544	1.2	0.173	[4]
rs954980	9:3761909	0.319	0.082	1.4	0.295	0.076	1.8	0.283	0.846	0.9	0.278	0.536	1.2	0.288	[1]
rs12378118	9:6033278	0.164	0.813	1.1	0.13	0.299	0.7	0.123	0.075	1.8	0.133	0.957	1.0	0.132	[1]
rs7873807	9:7582632	0.355	0.113	0.7	0.278	0.127	0.6	0.279	0.256	0.7	0.283	0.287	0.8	0.277	[1]
rs12378124	9:9668869	0.118	0.672	0.9	0.119	0.696	0.9	0.125	0.806	1.1	0.129	0.958	1.0	0.123	[1]
rs1412834	9:22110131	0.491	0.095	1.3	0.421	0.882	1.0	0.414	0.568	1.2	0.409	0.097	1.5	0.418	[3]
rs145503185	9:23455764	0.009	0.517	0.7	0.036	0.647	0.7	0.034	0.913	0.9	0.033	0.296	0.5	0.035	[5]
rs10989517	9:104222708	0.086	0.777	0.9	0.047	0.885	1.1	0.047	0.980	1.0	0.047	0.745	1.2	0.05	[1]
rs1056107	9:115087127	0.457	**0.028**	0.7	0.461	**0.048**	0.6	0.455	0.102	0.7	0.455	0.262	0.8	0.453	[1]
rs4979489	9:117768680	0.263	0.223	1.3	0.163	0.761	1.1	0.15	0.992	1.0	0.154	0.273	1.4	0.163	[1]
rs4836891	9:125273574	0.073	0.930	1.0	0.066	0.975	1.0	0.067	0.931	1.0	0.065	0.325	0.6	0.063	[1]
rs117146485	9:138824257	0.015	0.473	1.5	0.024	0.071	3.8	0.023	0.286	2.3	0.021	0.299	2.0	0.023	[8]
rs10795668	10:8701219	0.320	0.206	1.2	0.297	0.128	1.5	0.29	**0.023**	1.8	0.29	0.833	1.1	0.29	[22]
rs11255841	10:8739580	0.309	0.690	1.1	0.264	0.404	1.3	0.262	0.161	1.5	0.256	0.519	0.8	0.258	[2.6.21]
rs704017	10:80819132	0.438	0.572	0.9	0.439	0.983	1.0	0.441	0.731	1.1	0.438	0.998	1.0	0.441	[6]
rs11812485	10:83048037	0.046	0.687	1.1	0.054	0.477	1.5	0.057	0.532	1.4	0.056	0.481	0.7	0.054	[1]
rs1035209	10:101345366	0.195	0.483	1.1	0.195	0.905	1.0	0.204	0.374	0.8	0.195	0.133	1.5	0.197	[6.21]
rs2193352	10:101346609	0.195	0.478	1.1	0.195	0.888	1.0	0.204	0.374	0.8	0.196	0.131	1.5	0.197	[3]
rs11190164	10:101351704	0.281	0.132	1.3	0.258	0.826	1.1	0.268	0.822	0.9	0.264	0.096	1.5	0.263	[2]
rs11196171	10:114724473	0.242	0.831	1.0	0.238	0.547	0.8	0.223	0.773	0.9	0.227	0.721	0.9	0.231	[3]
rs1665650	10:118487100	0.259	0.396	0.9	0.25	0.077	0.6	0.245	0.264	0.7	0.248	0.936	1.0	0.249	[16]
rs307385	10:132250666	0.248	0.389	0.9	0.2	0.294	0.7	0.208	**0.017**	0.4	0.204	0.820	0.9	0.204	[10]
rs4086865	11:26012193	0.035	0.603	1.2	0.035	0.962	1.0	0.036	0.547	1.5	0.035	0.730	0.8	0.034	[1]
rs1535	11:61597972	0.35	0.206	0.8	0.317	0.901	1.0	0.317	0.392	0.8	0.318	0.305	0.8	0.315	[6]
rs57796856	11:74338355	0.48	0.185	1.2	0.499	0.224	1.3	0.488	0.553	1.2	0.487	0.928	1.0	0.482	[3]
rs3824999	11:74345550	0.476	0.248	0.8	0.488	0.215	0.7	0.496	0.503	0.9	0.496	0.817	1.0	0.497	[2.6.11.17]
rs11224824	11:101409138	0.113	0.722	1.1	0.091	0.983	1.0	0.086	0.730	1.2	0.088	0.910	1.0	0.085	[1]
rs7130173	11:111154072	0.27	0.820	1.0	0.314	0.124	1.6	0.302	0.890	1.0	0.304	0.749	0.9	0.31	[14]
rs3087967	11:111156836	0.268	0.946	1.0	0.307	0.199	1.5	0.295	0.868	1.0	0.3	0.640	0.9	0.304	[3.5]
rs3802842	11:111171709	0.269	0.728	1.1	0.308	0.222	1.5	0.294	0.792	1.1	0.3	0.883	1.0	0.305	[2.4.6.11.19]
rs6589219	11:111172911	0.281	0.586	1.1	0.324	0.220	1.5	0.308	0.611	1.2	0.315	0.775	0.9	0.319	[9]
rs7927484	11:123138960	0.187	0.643	1.1	0.262	0.352	1.3	0.255	0.694	1.1	0.256	0.763	1.1	0.265	[1]
rs10774214	12:4368352	0.381	0.074	0.7	0.314	0.801	0.9	0.316	**0.029**	0.5	0.308	0.313	0.8	0.321	[16]
rs3217810	12:4388271	0.121	0.349	1.3	0.112	0.900	1.1	0.104	0.775	1.1	0.109	0.061	1.9	0.116	[3.4.6]
rs3217901	12:4405389	0.381	0.194	1.2	0.356	0.391	1.3	0.343	0.890	1.0	0.345	0.143	1.4	0.354	[4.11]
rs10849438	12:6412036	0.141	0.627	1.1	0.133	0.471	1.3	0.129	0.130	0.5	0.127	0.570	0.8	0.128	[3]
rs34245511	12:50573433	0.362	0.913	1.0	0.428	0.948	1.0	0.44	0.362	0.8	0.435	0.963	1.0	0.435	[21]
rs11169552	12:51155663	0.249	**0.010**	1.7	0.175	**0.003**	2.8	0.174	0.074	1.8	0.171	0.374	1.3	0.168	[7]
rs11169572	12:51216890	0.387	0.934	1.0	0.487	0.824	0.9	0.483	0.794	0.9	0.484	0.483	1.2	0.488	[3]
rs10878977	12:69799690	0.297	0.361	1.2	0.248	0.153	1.6	0.256	0.315	1.3	0.255	0.168	1.4	0.254	[1]
rs597808	12:111973358	0.466	0.391	1.2	0.409	0.736	0.9	0.412	0.138	1.5	0.419	0.837	1.0	0.424	[3]
rs73208120	12:117747590	0.084	0.103	0.6	0.049	0.817	1.1	0.052	0.092	0.4	0.049	**0.030**	0.3	0.05	[6.11]
rs9579517	13:20177593	0.126	0.429	0.8	0.106	0.452	1.3	0.109	0.557	1.2	0.108	0.309	0.7	0.102	[10]
rs12427600	13:37460648	0.23	0.415	0.9	0.204	0.413	0.8	0.205	0.509	0.8	0.202	0.989	1.0	0.206	[3]
rs323473	13:105359644	0.039	0.676	1.2	0.032	0.401	1.7	0.034	0.758	1.2	0.034	0.817	0.9	0.03	[1]
rs9583269	13:109283030	0.342	0.449	0.9	0.322	0.766	0.9	0.331	0.058	0.6	0.32	0.468	0.8	0.333	[1]
rs7993934	13:111074915	0.362	0.298	1.2	0.377	0.825	0.9	0.372	0.266	1.4	0.381	0.072	1.5	0.385	[3]
rs8001158	13:111075658	0.361	0.273	1.2	0.377	0.842	0.9	0.372	0.258	1.4	0.38	0.067	1.6	0.385	[14]
rs1957032	14:32951556	0.447	0.230	0.8	0.475	0.269	0.7	0.476	0.506	0.9	0.481	0.977	1.0	0.478	[8]
rs17177725	14:37357412	0.102	0.878	0.9	0.061	0.505	1.4	0.062	0.989	1.0	0.058	0.826	0.9	0.058	[1]
rs28611105	14:51359658	0.203	0.671	1.1	0.22	0.562	1.2	0.212	0.762	1.1	0.205	0.157	1.4	0.225	[13]
rs4444235	14:54410919	0.493	0.656	0.9	0.446	0.619	0.9	0.453	0.576	0.9	0.445	0.822	0.9	0.446	[6.7.11]

rs1570405	14:54554234	0.319	0.667	1.1	0.341	0.750	0.9	0.329	0.210	1.4	0.329	0.472	1.2	0.336	[3]
rs17094983	14:59189361	0.121	0.828	1.0	0.15	0.085	1.8	0.15	0.167	0.6	0.143	0.240	1.4	0.152	[4.6]
rs12913832	15:28365618	0.364	0.749	0.9	0.41	0.574	1.2	0.407	0.424	1.3	0.407	0.604	0.9	0.404	[1]
rs12916300	15:28410491	0.344	0.965	1.0	0.439	0.930	1.0	0.435	0.264	1.4	0.437	0.440	0.8	0.43	[1]
rs4779584	15:32994756	0.205	0.686	0.9	0.165	0.553	1.2	0.16	0.405	0.8	0.163	0.965	1.0	0.162	[4.11.19.22]
rs58658771	15:33001734	0.185	0.518	0.9	0.155	0.376	1.3	0.153	0.360	0.7	0.153	0.567	0.8	0.151	[18.24]
rs2293582	15:33010412	0.204	0.568	0.9	0.179	0.356	1.3	0.18	0.772	0.9	0.179	0.243	0.7	0.173	[2]
rs73376930	15:33012502	0.207	0.627	0.9	0.179	0.312	1.4	0.18	0.853	0.9	0.179	0.289	0.7	0.174	[3.6.21]
rs17816465	15:33156386	0.178	0.521	0.9	0.162	0.543	0.8	0.161	0.885	1.0	0.164	0.279	0.7	0.155	[3]
rs2439411	15:66983982	0.215	0.898	1.0	0.226	0.924	1.0	0.222	0.254	1.4	0.23	0.690	0.9	0.217	[14]
rs4776316	15:67007813	0.276	0.353	1.2	0.29	0.971	1.0	0.28	0.556	1.2	0.287	0.885	1.0	0.283	[3]
rs11853542	15:88052767	0.141	0.708	1.1	0.133	0.786	0.9	0.125	0.505	0.8	0.131	0.700	1.1	0.128	[1]
rs16944613	15:91139098	0.242	0.925	1.0	0.232	0.286	0.7	0.236	0.940	1.0	0.239	0.756	0.9	0.232	[1]
rs7495132	15:91172901	0.096	0.236	0.7	0.108	0.199	0.6	0.115	0.248	0.6	0.111	0.068	0.5	0.107	[3]
rs476605	16:5429577	0.374	0.830	1.0	0.396	0.918	1.0	0.397	0.093	1.7	0.399	0.259	0.7	0.384	[1]
rs79900961	16:9297812	0.015	0.796	0.9	0.017	0.550	0.5	0.016	0.238	2.5	0.019	0.433	0.5	0.016	[2]
rs35874699	16:26539262	0.429	0.141	0.8	0.335	0.156	0.7	0.343	0.328	0.8	0.339	0.575	0.9	0.34	[1]
rs9939049	16:68812301	0.296	0.232	0.8	0.267	0.844	0.9	0.276	0.817	0.9	0.274	0.357	0.8	0.275	[3]
rs9929218	16:68820946	0.294	0.200	0.8	0.269	0.877	1.0	0.279	0.711	0.9	0.276	0.334	0.8	0.279	[6.7]
rs61336918	16:80007266	0.296	0.216	0.8	0.287	0.486	0.8	0.29	**0.025**	0.5	0.278	0.623	0.9	0.291	[3]
rs16941835	16:86695720	0.211	0.597	1.1	0.168	0.914	1.0	0.168	0.391	1.3	0.17	0.731	1.1	0.167	[2]
rs899244	16:86700030	0.222	0.214	1.3	0.179	0.784	1.1	0.178	0.132	1.6	0.179	0.261	1.4	0.177	[3]
rs35467001	17:71380062	0.067	0.188	1.5	0.064	0.800	0.9	0.064	0.432	0.7	0.063	0.366	1.5	0.062	[25]
rs75954926	17:81061048	0.339	0.303	0.8	0.337	0.788	1.1	0.344	0.062	0.6	0.333	0.107	0.7	0.332	[3]
rs76409885	17:81064661	0.371	0.544	0.9	0.374	0.549	1.2	0.371	0.151	0.7	0.364	0.138	0.7	0.362	[14]
rs12604637	18:8556716	0.103	0.380	1.3	0.082	0.255	1.8	0.076	0.537	1.4	0.077	0.619	1.2	0.083	[1]
rs674617	18:9701782	0.101	0.949	1.0	0.054	0.799	1.2	0.058	0.802	1.1	0.054	0.381	0.6	0.053	[1]
rs35505947	18:13954109	0.142	0.630	1.1	0.136	0.738	1.1	0.137	0.537	1.2	0.136	0.140	1.6	0.141	[1]
rs6507874	18:46448805	0.458	**0.050**	0.7	0.39	0.226	0.7	0.414	0.055	0.6	0.412	0.247	0.8	0.414	[18.24]
rs12953717	18:46453929	0.448	0.332	0.9	0.463	0.720	0.9	0.478	0.240	0.8	0.475	0.964	1.0	0.479	[1]
rs1262463	18:52954421	0.062	0.141	0.6	0.057	0.210	0.5	0.061	0.265	0.6	0.062	0.357	0.7	0.062	[1]
rs285245	19:16420817	0.1	0.418	1.2	0.121	0.871	0.9	0.113	0.770	1.1	0.121	0.978	1.0	0.117	[3]
rs28570619	19:33517152	0.063	0.496	0.8	0.075	0.978	1.0	0.079	0.155	0.4	0.077	0.957	1.0	0.079	[14]
rs17841839	19:33517201	0.063	0.496	0.8	0.075	0.977	1.0	0.079	0.155	0.4	0.077	0.957	1.0	0.08	[5]
rs73039434	19:33524919	0.061	0.891	1.0	0.089	0.717	1.2	0.095	0.713	1.2	0.094	0.605	0.8	0.094	[3]
rs13343954	19:33527888	0.149	0.196	0.7	0.138	0.220	0.6	0.146	0.611	0.8	0.146	0.930	1.0	0.148	[21]
rs10411210	19:33532300	0.097	0.533	0.9	0.125	0.384	0.7	0.132	0.706	1.2	0.135	0.999	1.0	0.137	[6.7]
rs28541881	19:34768590	0.05	0.159	1.8	0.044	0.560	1.5	0.043	0.484	1.5	0.044	0.180	2.0	0.046	[1]
rs2965280	19:34917700	0.048	0.413	1.3	0.05	0.723	1.2	0.05	0.680	1.2	0.05	0.521	1.4	0.051	[1]
rs12979278	19:49218602	0.491	0.241	0.8	0.48	0.127	0.6	0.479	0.339	0.8	0.488	0.691	0.9	0.492	[3]
rs1654668	19:55174213	0.442	0.857	1.0	0.453	0.243	0.7	0.447	0.973	1.0	0.453	0.300	1.3	0.449	[1]
rs9676308	19:57579077	0.034	0.215	0.5	0.025	0.936	1.1	0.026	0.551	0.7	0.024	0.308	0.5	0.025	[1]
rs961253	20:6404281	0.361	0.065	1.4	0.328	0.364	1.3	0.327	0.714	1.1	0.323	0.505	1.2	0.323	[3.6.7.9.11]
rs1015563	20:6690101	0.325	0.778	1.0	0.31	0.800	1.1	0.314	0.685	0.9	0.311	0.895	1.0	0.313	[2]
rs6085661	20:6693128	0.353	0.468	0.9	0.343	0.497	0.8	0.347	0.330	0.8	0.342	0.834	1.0	0.344	[3.14]
rs4813802	20:6699595	0.318	0.915	1.0	0.307	0.822	1.1	0.307	0.622	0.9	0.307	0.692	1.1	0.309	[4.6]
rs6086194	20:7749391	0.264	0.530	0.9	0.299	0.375	0.8	0.302	0.690	0.9	0.297	0.060	0.6	0.304	[14]
rs2423279	20:7812350	0.271	0.160	0.8	0.313	0.264	0.7	0.318	0.373	0.8	0.31	**0.028**	0.6	0.316	[16]
rs8114643	20:7833046	0.154	0.488	0.9	0.157	0.194	0.6	0.158	0.930	1.0	0.161	**0.034**	0.5	0.158	[5]
rs2179593	20:42660286	0.283	0.766	1.1	0.272	0.565	1.2	0.276	0.673	1.1	0.275	0.997	1.0	0.272	[3]
rs4811050	20:48980670	0.199	0.678	0.9	0.151	0.562	0.8	0.147	0.741	0.9	0.145	0.943	1.0	0.149	[3]
rs1810501	20:49057534	0.449	0.677	0.9	0.484	0.621	0.9	0.491	0.232	0.7	0.496	0.332	0.8	0.493	[14]

rs4925386	20:60921044	0.332	**0.015**	0.7	0.314	0.161	0.7	0.329	**0.012**	0.5	0.32	0.465	0.9	0.324	[7]
rs1741640	20:60932414	0.243	**0.022**	0.7	0.25	0.369	0.8	0.26	0.108	0.6	0.257	0.794	0.9	0.26	[3]
rs2427308	20:60969451	0.233	0.051	0.7	0.24	0.207	0.7	0.248	0.079	0.6	0.244	0.790	1.1	0.249	[2.6.21]
rs2427313	20:60970675	0.299	0.052	0.7	0.323	0.057	0.6	0.338	0.055	0.6	0.333	0.946	1.0	0.339	[14]
rs3787089	20:62316630	0.294	0.312	1.2	0.263	0.603	1.2	0.267	0.641	1.1	0.255	0.812	0.9	0.265	[3]
rs12627687	21:35517484	0.202	0.250	1.3	0.163	0.425	1.4	0.164	0.535	1.3	0.168	0.581	1.2	0.163	[1]
rs5753618	22:31838518	0.222	0.197	0.7	0.166	0.151	0.5	0.169	0.096	0.5	0.173	0.643	0.9	0.169	[1]
rs736037	22:45724999	0.275	0.553	1.1	0.301	0.392	1.3	0.308	0.306	1.3	0.309	0.742	1.1	0.307	[13]

1. Hofer, P.; Hagmann, M.; Brezina, S.; Dolejsi, E.; Mach, K.; Leeb, G.; Baierl, A.; Buch, S.; Sutterlüty-Fall, H.; Karner-Hanusch, J.; et al. Bayesian and frequentist analysis of an Austrian genome-wide association study of colorectal cancer and advanced adenomas. *Oncotarget* **2017**, *8*, 98623–98634, doi:10.18632/oncotarget.21697.
2. Al-Tassan, N.A.; Whiffin, N.; Hosking, F.J.; Palles, C.; Farrington, S.M.; Dobbins, S.E.; Harris, R.; Gorman, M.; Tenesa, A.; Meyer, B.F.; et al. A new GWAS and meta-analysis with 1000Genomes imputation identifies novel risk variants for colorectal cancer. *Sci. Rep.* **2015**, *5*, 10442, doi:10.1038/srep10442.
3. Law, P.J.; Timofeeva, M.; Fernandez-Rozadilla, C.; Broderick, P.; Studd, J.; Fernandez-Tajes, J.; Farrington, S.; Svinti, V.; Palles, C.; Orlando, G.; et al. Association analyses identify 31 new risk loci for colorectal cancer susceptibility. *Nat. Commun.* **2019**, *10*, 1–15, doi:10.1038/s41467-019-09775-w.
4. Peters, U.; Jiao, S.; Schumacher, F.R.; Hutter, C.M.; Aragaki, A.K.; Baron, J.A.; Berndt, S.I.; Bézieau, S.; Brenner, H.; Butterbach, K.; et al. Identification of Genetic Susceptibility Loci for Colorectal Tumors in a Genome-Wide Meta-analysis. *Gastroenterology* **2013**, *144*, 799-807.e24, doi:10.1053/j.gastro.2012.12.020.
5. Kachuri, L.; Graff, R.E.; Smith-Byrne, K.; Meyers, T.J.; Rashkin, S.R.; Ziv, E.; Witte, J.S.; Johansson, M. Pan-cancer analysis demonstrates that integrating polygenic risk scores with modifiable risk factors improves risk prediction. *Nat. Commun.* **2020**, *11*, 6084, doi:10.1038/s41467-020-19600-4.
6. Schmit, S.L.; Edlund, C.K.; Schumacher, F.R.; Gong, J.; Harrison, T.A.; Huyghe, J.R.; Qu, C.; Melas, M.; Van Den Berg, D.J.; Wang, H.; et al. Novel Common Genetic Susceptibility Loci for Colorectal Cancer. *JNCI J. Natl. Cancer Inst.* **2019**, *111*, 146–157, doi:10.1093/jnci/djy099.
7. Houlston, R.S.; Cheadle, J.; Dobbins, S.E.; Tenesa, A.; Jones, A.M.; Howarth, K.; Spain, S.L.; Broderick, P.; Domingo, E.; Farrington, S.; et al. Meta-analysis of three genome-wide association studies identifies susceptibility loci for colorectal cancer at 1q41, 3q26.2, 12q13.13 and 20q13.33. *Nat. Genet.* **2010**, *42*, 973–977, doi:10.1038/ng.670.
8. Brandes, N.; Linial, N.; Linial, M. Genetic association studies of alterations in protein function expose recessive effects on cancer predisposition. *Sci. Rep.* **2021**, *11*, 14901, doi:10.1038/s41598-021-94252-y.
9. Tanskanen, T.; van den Berg, L.; Välimäki, N.; Aavikko, M.; Ness-Jensen, E.; Hveem, K.; Wettergren, Y.; Bexe Lindskog, E.; Tõnisson, N.; Metspalu, A.; et al. Genome-wide association study and meta-analysis in Northern European populations replicate multiple colorectal cancer risk loci. *Int. J. cancer* **2018**, *142*, 540–546, doi:10.1002/ijc.31076.
10. Nazarian, A.; Kulminski, A.M. Genome-Wide Analysis of Sex Disparities in the Genetic Architecture of Lung and Colorectal Cancers. *Genes (Basel).* **2021**, *12*, doi:10.3390/genes12050686.
11. Schumacher, F.R.; Schmit, S.L.; Jiao, S.; Edlund, C.K.; Wang, H.; Zhang, B.; Hsu, L.; Huang, S.-C.; Fischer, C.P.; Harju, J.F.; et al. Genome-wide association study of colorectal cancer identifies six new susceptibility loci. *Nat. Commun.* **2015**, *6*, 7138, doi:10.1038/ncomms8138.
12. Fernandez-Rozadilla, C.; Cazier, J.-B.; Tomlinson, I.P.; Carvajal-Carmona, L.G.; Palles, C.; Lamas, M.J.; Baiget, M.; López-Fernández, L.A.; Brea-Fernández, A.; Abulí, A.; et al. A colorectal cancer genome-wide association study in a Spanish cohort identifies two variants associated with colorectal cancer risk at 1p33 and 8p12. *BMC Genomics* **2013**, *14*, 55, doi:10.1186/1471-2164-14-55.
13. Huyghe, J.R.; Harrison, T.A.; Bien, S.A.; Hampel, H.; Figueiredo, J.C.; Schmit, S.L.; Conti, D. V.; Chen, S.; Qu, C.; Lin, Y.; et al. Genetic architectures of proximal and distal colorectal cancer are partly distinct. *Gut* **2021**, *70*, 1325–1334, doi:10.1136/gutjnl-2020-321534.
14. Sakaue, S.; Kanai, M.; Tanigawa, Y.; Karjalainen, J.; Kurki, M.; Koshiba, S.; Narita, A.; Konuma, T.; Yamamoto, K.; Akiyama, M.; et al. A cross-population atlas of genetic associations for 220 human phenotypes. *Nat. Genet.* **2021**, *53*, 1415–1424, doi:10.1038/s41588-021-00931-x.
15. Schmit, S.L.; Schumacher, F.R.; Edlund, C.K.; Conti, D. V; Raskin, L.; Lejbkowicz, F.; Pinchev, M.; Rennert, H.S.; Jenkins, M.A.; Hopper, J.L.; et al. A novel colorectal cancer risk locus at 4q32.2 identified from an international genome-wide association study. *Carcinogenesis* **2014**, *35*, 2512–2519, doi:10.1093/carcin/bgu148.
16. Jia, W.-H.; Zhang, B.; Matsuo, K.; Shin, A.; Xiang, Y.-B.; Jee, S.H.; Kim, D.-H.; Ren, Z.; Cai, Q.; Long, J.; et al. Genome-wide association analyses in East Asians identify new susceptibility loci for colorectal cancer. *Nat. Genet.* **2013**, *45*, 191–196, doi:10.1038/ng.2505.
17. Dunlop, M.G.; Dobbins, S.E.; Farrington, S.M.; Jones, A.M.; Palles, C.; Whiffin, N.; Tenesa, A.; Spain, S.; Broderick, P.; Ooi, L.-Y.; et al. Common variation near CDKN1A, POLD3 and SHROOM2 influences colorectal cancer risk. *Nat. Genet.* **2012**, *44*, 770–776, doi:10.1038/ng.2293.
18. Rashkin, S.R.; Graff, R.E.; Kachuri, L.; Thai, K.K.; Alexeeff, S.E.; Blatchins, M.A.; Cavazos, T.B.; Corley, D.A.; Emami, N.C.; Hoffman, J.D.; et al. Pan-cancer study detects genetic risk variants and shared genetic basis in two large cohorts. *Nat. Commun.* **2020**, *11*, 4423, doi:10.1038/s41467-020-18246-6.
19. Peters, U.; Hutter, C.M.; Hsu, L.; Schumacher, F.R.; Conti, D. V; Carlson, C.S.; Edlund, C.K.; Haile, R.W.; Gallinger, S.; Zanke, B.W.; et al. Meta-analysis of new genome-wide association studies of colorectal cancer risk. *Hum. Genet.* **2012**, *131*, 217–234, doi:10.1007/s00439-011-1055-0.
20. Tomlinson, I.; Webb, E.; Carvajal-Carmona, L.; Broderick, P.; Kemp, Z.; Spain, S.; Penegar, S.; Chandler, I.; Gorman, M.; Wood, W.; et al. A genome-wide association scan of tag SNPs identifies a susceptibility variant for colorectal cancer at 8q24.21. *Nat. Genet.* **2007**, *39*, 984–988, doi:10.1038/ng2085.
21. Whiffin, N.; Hosking, F.J.; Farrington, S.M.; Palles, C.; Dobbins, S.E.; Zgaga, L.; Lloyd, A.; Kinnersley, B.; Gorman, M.; Tenesa, A.; et al. Identification of susceptibility loci for colorectal

cancer in a genome-wide meta-analysis. *Hum. Mol. Genet.* **2014**, *23*, 4729–4737, doi:10.1093/hmg/ddu177.
22. Tomlinson, I.P.M.; Webb, E.; Carvajal-Carmona, L.; Broderick, P.; Howarth, K.; Pittman, A.M.; Spain, S.; Lubbe, S.; Walther, A.; Sullivan, K.; et al. A genome-wide association study identifies colorectal cancer susceptibility loci on chromosomes 10p14 and 8q23.3. *Nat. Genet.* **2008**, *40*, 623–630, doi:10.1038/ng.111.
23. Tenesa, A.; Farrington, S.M.; Prendergast, J.G.D.; Porteous, M.E.; Walker, M.; Haq, N.; Barnetson, R.A.; Theodoratou, E.; Cetnarskyj, R.; Cartwright, N.; et al. Genome-wide association scan identifies a colorectal cancer susceptibility locus on 11q23 and replicates risk loci at 8q24 and 18q21. *Nat. Genet.* **2008**, *40*, 631–637, doi:10.1038/ng.133.
24. Zhou, W.; Nielsen, J.B.; Fritsche, L.G.; Dey, R.; Gabrielsen, M.E.; Wolford, B.N.; LeFaive, J.; VandeHaar, P.; Gagliano, S.A.; Gifford, A.; et al. Efficiently controlling for case-control imbalance and sample relatedness in large-scale genetic association studies. *Nat. Genet.* **2018**, *50*, 1335–1341, doi:10.1038/s41588-018-0184-y.
25. Matejcic, M.; Shaban, H.A.; Quintana, M.W.; Schumacher, F.R.; Edlund, C.K.; Naghi, L.; Pai, R.K.; Haile, R.W.; Levine, A.J.; Buchanan, D.D.; et al. Rare Variants in the DNA Repair Pathway and the Risk of Colorectal Cancer. *Cancer Epidemiol. biomarkers Prev. a Publ. Am. Assoc. Cancer Res. cosponsored by Am. Soc. Prev. Oncol.* **2021**, *30*, 895–903, doi:10.1158/1055-9965.EPI-20-1457.

Supplementary Table S2: Sensitivity analyses of used instruments in Mendelian Randomization analyses.

Exposure	Number of SNPs	Explained variance (R^2)	F-statistic	I^2
BMI (GIANT)	360	0.0368	72.33	0.9867
BMI (MRC-IEU)	314	0.0402	61.54	0.983
BMI (MRC-IEU2)	312	0.0414	62.91	0.9834
BMI (Hoffmann 2018)	102	0.0199	62.76	0.9834
Total cholesterol (GLCL)	59	0.0557	187.30	0.993
HDL cholesterol (GLCL)	58	0.0415	139.61	0.9921
LDL cholesterol (GLCL)	56	0.064	211.34	0.9943
Triglycerides (GLGC)	34	0.0354	191.98	0.9946
Basal metabolic rate (MRC-IEU)	378	0.066	85.02	0.9874
Body fat percentage (MRC-IEU)	287	0.0365	60.01	0.9826
Waist circumference (MRC-IEU)	250	0.0293	55.82	0.9807
Standing height (MRC-IEU)	480	0.1504	170.22	0.9931
Actinobacteria	39	0.0422	20.28	0.9497
Bacteroidetes	40	0.0413	19.33	0.9451
Cyanobacteria	34	0.0349	19.07	0.9474
Euryarchaeota	26	0.0271	19.26	0.946
Firmicutes	45	0.0472	19.76	0.9473
Lentisphaerae	40	0.0411	19.21	0.9451
Proteobacteria	34	0.0342	18.73	0.9453
Tenericutes	36	0.0371	19.23	0.9454
Verrucomicrobia	32	0.0337	19.58	0.9466

Supplementary Table S3: Results of Mendelian Randomization analyses using modifiable risk factors as exposures. SE, standard error of beta value. Heterogeneity, p-value of Q-test

		Inverse variance weighted			Weighted median			MR Egger				
**Exposure**	**Outcome**	**Beta**	**SE**	**P-value**	**Beta**	**SE**	**P-value**	**Beta**	**SE**	**P-value**	**Heterogeneity**	**Pleiotropy**
BMI (GIANT)	Colorectal cancer	-0.33	0.59	0.5749	0.59	0.96	0.5374	2.81	1.50	0.0613	0.7749	0.0231
BMI (MRC-IEU)		0.01	0.61	0.9882	0.69	0.97	0.4801	2.10	1.76	0.2332	0.5673	0.2062
BMI (MRC-IEU2)		-0.23	0.60	0.6997	0.60	0.94	0.5263	1.94	1.73	0.2623	0.5472	0.1813
BMI (Hoffmann 2018)		0.80	0.79	0.3155	1.16	1.23	0.3465	3.64	2.33	0.1208	0.5298	0.1964
Total cholesterol (GLCL)		0.07	0.45	0.8715	0.13	0.69	0.8454	0.80	0.70	0.2570	0.4593	0.1788
HDL cholesterol (GLCL)		0.32	0.53	0.5390	0.09	0.82	0.9074	0.58	0.93	0.5386	0.8881	0.7433
LDL cholesterol (GLCL)		0.51	0.46	0.2619	0.70	0.61	0.2514	0.88	0.65	0.1804	0.1176	0.4280
Triglycerides (GLGC)		0.14	0.61	0.8217	-0.24	0.89	0.7891	0.49	0.94	0.6078	0.9977	0.6288
Basal metabolic rate (MRC-IEU)		-0.11	0.72	0.8729	-0.38	1.19	0.7482	0.04	1.78	0.9839	0.8987	0.9261
Body fat percentage (MRC-IEU)		0.22	0.82	0.7857	-0.37	1.21	0.7607	0.24	2.78	0.9302	0.4921	0.9936
Waist circumference (MRC-IEU)		-0.16	0.79	0.8418	-0.31	1.22	0.7957	0.49	2.55	0.8477	0.6763	0.7896
Standing height (MRC-IEU)		-0.29	0.47	0.5419	-0.69	0.75	0.3605	0.69	0.93	0.4550	0.7029	0.2206
BMI (GIANT)	Right colon cancer	0.24	0.96	0.8033	1.10	1.72	0.5227	3.44	2.42	0.1558	0.9939	0.1500
BMI (MRC-IEU)		0.30	1.00	0.7624	1.23	1.52	0.4201	1.51	2.90	0.6030	0.9481	0.6577
BMI (MRC-IEU2)		0.32	0.99	0.7480	0.85	1.59	0.5917	0.80	2.84	0.7782	0.8800	0.8561
BMI (Hoffmann 2018)		0.16	1.31	0.9044	0.99	1.94	0.6082	5.10	3.79	0.1819	0.9899	0.1682
Total cholesterol (GLCL)		0.21	0.71	0.7633	0.12	1.07	0.9122	0.93	1.07	0.3896	0.9614	0.3765
HDL cholesterol (GLCL)		0.69	0.86	0.4259	0.81	1.29	0.5309	0.42	1.52	0.7831	0.8954	0.8333
LDL cholesterol (GLCL)		0.80	0.64	0.2125	0.99	0.89	0.2646	0.81	0.87	0.3555	0.8462	0.9841
Triglycerides (GLGC)		1.05	1.01	0.2965	-0.29	1.53	0.8513	2.60	1.58	0.1102	0.9067	0.2138
Basal metabolic rate (MRC-IEU)		-0.05	1.18	0.9673	0.85	1.93	0.6612	1.25	2.91	0.6666	0.9914	0.6245
Body fat percentage (MRC-IEU)		-1.36	1.35	0.3109	-1.28	2.07	0.5360	-0.31	4.60	0.9467	0.9805	0.8101
Waist circumference (MRC-IEU)		-0.16	1.29	0.9028	0.87	2.09	0.6779	3.04	4.14	0.4632	0.9141	0.4168
Standing height (MRC-IEU)		-1.04	0.77	0.1742	-0.16	1.29	0.9044	1.41	1.51	0.3497	0.9576	0.0593
BMI (GIANT)	Left colon cancer	0.04	0.92	0.9614	0.43	1.48	0.7735	2.66	2.35	0.2575	0.8306	0.2266
BMI (MRC-IEU)		-0.36	0.95	0.7028	-1.13	1.47	0.4428	0.59	2.80	0.8336	0.7815	0.7187
BMI (MRC-IEU2)		-0.70	0.93	0.4541	-1.11	1.49	0.4577	0.23	2.74	0.9334	0.9054	0.7189
BMI (Hoffmann 2018)		1.41	1.24	0.2567	0.74	1.96	0.7081	-0.53	3.66	0.8858	0.6913	0.5756
Total cholesterol (GLCL)		0.74	0.72	0.3064	1.71	1.03	0.0969	2.36	1.12	0.0395	0.6872	0.0633
HDL cholesterol (GLCL)		-0.35	0.83	0.6718	-1.52	1.21	0.2098	-0.25	1.47	0.8641	0.3448	0.9342
LDL cholesterol (GLCL)		1.56	0.64	0.0148	1.39	0.87	0.1114	1.06	0.89	0.2372	0.9654	0.4231
Triglycerides (GLGC)		1.05	0.96	0.2740	1.31	1.36	0.3382	0.64	1.51	0.6755	0.9396	0.7243
Basal metabolic rate (MRC-IEU)		-1.56	1.12	0.1628	-1.65	1.80	0.3591	0.89	2.80	0.7514	0.6322	0.3394
Body fat percentage (MRC-IEU)		1.13	1.27	0.3741	-0.29	1.92	0.8791	0.50	4.35	0.9089	0.5912	0.8788
Waist circumference (MRC-IEU)		0.17	1.23	0.8868	-0.42	1.87	0.8237	-0.34	4.04	0.9327	0.7936	0.8933
Standing height (MRC-IEU)		-0.69	0.73	0.3476	-0.47	1.20	0.6963	-1.33	1.45	0.3594	0.6236	0.6084
BMI (GIANT)	Rectal cancer	-0.69	0.84	0.4130	-0.70	1.51	0.6431	2.15	2.13	0.3131	0.3721	0.1480
BMI (MRC-IEU)		0.36	0.86	0.6800	0.51	1.36	0.7083	3.49	2.49	0.1614	0.3351	0.1800
BMI (MRC-IEU2)		-0.23	0.87	0.7932	0.48	1.30	0.7120	3.44	2.50	0.1688	0.1614	0.1174
BMI (Hoffmann 2018)		0.72	1.12	0.5183	0.57	1.75	0.7462	8.69	3.28	0.0094	0.8215	0.0112
Total cholesterol (GLCL)		0.57	0.63	0.3631	0.66	0.96	0.4954	0.70	0.95	0.4653	0.6331	0.8571
HDL cholesterol (GLCL)		0.81	0.74	0.2726	0.52	1.21	0.6674	1.00	1.31	0.4513	0.6994	0.8681
LDL cholesterol (GLCL)		0.82	0.58	0.1571	0.97	0.83	0.2416	1.00	0.82	0.2322	0.4488	0.7677
Triglycerides (GLGC)		0.11	0.87	0.8974	-0.69	1.23	0.5761	0.15	1.35	0.9098	0.9642	0.9673
Basal metabolic rate (MRC-IEU)		0.29	1.02	0.7788	0.01	1.65	0.9970	0.21	2.51	0.9329	0.9827	0.9744
Body fat percentage (MRC-IEU)		-0.46	1.15	0.6882	-1.09	1.79	0.5409	1.46	3.88	0.7061	0.4849	0.6034

Waist circumference (MRC-IEU)	-0.79	1.11	0.4769	-0.44	1.70	0.7959	-0.99	3.56	0.7813	0.4590	0.9531
Standing height (MRC-IEU)	-0.21	0.66	0.7497	0.02	1.05	0.9850	0.11	1.31	0.9321	0.5470	0.7750

Supplementary Table S4: Results of Mendelian Randomization analyses using bacterial phyla as exposures. SE, standard error of beta value. Heterogeneity, p-value of Q-test.

		Inverse variance weighted			Weighted median			MR Egger				
**Exposure**	**Outcome**	**Beta**	**SE**	**P-value**	**Beta**	**SE**	**P-value**	**Beta**	**SE**	**P-value**	**Heterogeneity**	**Pleiotropy**
Actinobacteria		-0.32	0.53	0.5501	-0.28	0.70	0.6863	-0.16	1.57	0.9202	0.8971	0.9140
Bacteroidetes		-0.56	0.57	0.3220	-0.49	0.81	0.5437	1.43	1.63	0.3887	0.4567	0.2037
Cyanobacteria		-0.86	0.39	0.0299	-0.47	0.55	0.3955	-1.64	1.28	0.2074	0.3770	0.5214
Euryarchaeota		-0.01	0.34	0.9841	-0.30	0.43	0.4752	-0.22	1.30	0.8675	0.2179	0.8667
Firmicutes	Colorectal cancer	0.20	0.61	0.7423	-0.13	0.80	0.8689	3.58	1.66	0.0364	0.0336	0.0347
Lentisphaerae		0.14	0.32	0.6515	0.39	0.44	0.3742	1.42	1.19	0.2395	0.2510	0.2719
Proteobacteria		-0.97	0.63	0.1254	-1.02	0.88	0.2476	-1.85	2.78	0.5096	0.7339	0.7454
Tenericutes		0.50	0.46	0.2826	0.78	0.61	0.2051	1.42	1.57	0.3724	0.4475	0.5427
Verrucomicrobia		-0.19	0.51	0.7040	0.07	0.71	0.9165	0.16	1.41	0.9122	0.9521	0.7928
Actinobacteria		-0.22	0.88	0.8016	0.35	1.25	0.7777	0.50	2.48	0.8409	0.8186	0.7570
Bacteroidetes		-0.35	0.92	0.7073	0.83	1.35	0.5376	2.76	2.67	0.3066	0.7316	0.2215
Cyanobacteria		-0.99	0.67	0.1390	0.00	0.91	0.9977	-0.49	2.14	0.8209	0.2652	0.8075
Euryarchaeota		0.12	0.57	0.8297	-0.45	0.74	0.5411	-2.40	2.11	0.2681	0.1931	0.2284
Firmicutes	Right colon cancer	-0.28	0.94	0.7679	0.79	1.33	0.5518	1.83	2.61	0.4871	0.1214	0.3906
Lentisphaerae		0.38	0.51	0.4577	0.17	0.72	0.8154	3.18	1.92	0.1062	0.3578	0.1393
Proteobacteria		-0.41	1.02	0.6917	-0.58	1.39	0.6794	-4.99	4.55	0.2817	0.9615	0.3096
Tenericutes		0.59	0.77	0.4400	0.61	1.03	0.5547	4.35	2.64	0.1078	0.8727	0.1451
Verrucomicrobia		0.32	0.86	0.7098	0.34	1.14	0.7659	-1.22	2.52	0.6324	0.9720	0.5213
Actinobacteria		-0.39	0.84	0.6447	-0.10	1.23	0.9336	-1.14	2.52	0.6521	0.4657	0.7507
Bacteroidetes		-0.11	1.00	0.9094	-0.62	1.36	0.6496	1.05	3.00	0.7276	0.1161	0.6820
Cyanobacteria		-1.66	0.68	0.0140	-1.19	0.92	0.1927	-1.53	2.16	0.4840	0.0973	0.9478
Euryarchaeota		-0.20	0.49	0.6784	-0.83	0.67	0.2183	0.99	1.79	0.5850	0.7105	0.4955
Firmicutes	Left colon cancer	0.96	1.02	0.3452	1.56	1.22	0.2021	6.40	2.82	0.0282	0.0107	0.0456
Lentisphaerae		0.31	0.47	0.5154	0.66	0.68	0.3339	-0.35	1.78	0.8461	0.4094	0.7058
Proteobacteria		-0.35	1.18	0.7670	-0.25	1.51	0.8667	1.17	5.35	0.8277	0.0602	0.7721
Tenericutes		1.05	0.78	0.1791	1.14	1.06	0.2806	2.84	2.50	0.2650	0.2099	0.4569
Verrucomicrobia		-0.60	0.78	0.4424	-0.46	1.09	0.6715	1.41	2.13	0.5116	0.9927	0.3176
Actinobacteria		-0.69	0.76	0.3639	-0.72	1.08	0.5033	0.73	2.23	0.7448	0.7367	0.5027
Bacteroidetes		-0.96	0.80	0.2296	-0.66	1.16	0.5713	-0.98	2.31	0.6737	0.5496	0.9905
Cyanobacteria		-0.82	0.55	0.1346	-0.29	0.78	0.7128	-1.91	1.75	0.2831	0.5902	0.5151
Euryarchaeota		0.37	0.44	0.4067	0.12	0.61	0.8488	-0.04	1.65	0.9817	0.8829	0.8012
Firmicutes	Rectal cancer	-0.23	0.70	0.7417	-0.19	1.04	0.8539	1.63	1.94	0.4070	0.5851	0.3101
Lentisphaerae		-0.05	0.45	0.9137	0.47	0.59	0.4284	0.89	1.72	0.6072	0.1788	0.5738
Proteobacteria		-0.36	0.89	0.6821	0.05	1.23	0.9656	2.31	3.79	0.5460	0.5349	0.4729
Tenericutes		0.56	0.64	0.3820	1.20	0.88	0.1753	1.62	2.14	0.4555	0.8548	0.6090
Verrucomicrobia		-0.07	0.70	0.9185	-0.59	1.04	0.5697	-1.01	1.92	0.6011	0.6890	0.6017
